# The spatiotemporal distribution of potential saxitoxin-producing cyanobacteria in western Lake Erie

**DOI:** 10.1016/j.jglr.2024.102342

**Published:** 2024-03-30

**Authors:** Callie Nauman, Keara Stanislawczyk, Laura A. Reitz, Justin D. Chaffin

**Affiliations:** a Biological Sciences, Bowling Green State University, Bowling Green, OH, USA; b F.T Stone Laboratory, The Ohio State University, 878 Bayview Ave. Put-in-Bay, OH 43456, USA

**Keywords:** Benthic algae, Eutrophication, Harmful algal blooms, Saxitoxin

## Abstract

Cyanobacterial blooms in the western basin of Lake Erie have been well studied with a focus on planktonic *Microcystis* and the cyanotoxin microcystin, but recent research has shown that blooms are not entirely *Microcystis*. Previous studies have documented other taxa in blooms capable of producing other cyanotoxins. Furthermore, benthic cyanobacteria have historically been overlooked in Lake Erie. Saxitoxin is a cyanotoxin of emerging concern in freshwater, and the *sxtA* gene which encodes its production has been found in the Maumee River and central basin of Lake Erie. Collectively, these points indicated that saxitoxin-producing cyanobacteria may also occur in the western basin. We utilized three sources of data to determine the spatial and temporal distribution of potential saxitoxin-producing cyanobacteria in the water column (years 2018–2022) and deployed nutrient diffusing substrata (NDS) to determine the impact of nutrients, depth, and season on potential-STX producing benthic cyanobacteria (years 2018 & 2019). The water column datasets showed that “hotspots” of *sxtA* lasted only a few weeks. *sxtA* gene copies per mL did not correlate with *Dolichospermum* or *Aphanizomenon* biovolume, which have been associated with *sxtA* elsewhere. In the NDS, saxitoxin (ng/cm^2^) and cyanobacteria chlorophyll were inversely correlated with the highest saxitoxin in September and at the deeper depth, whereas cyanobacteria chlorophyll was highest during June and at the shallower depth. This research suggests continued monitoring is needed to determine drivers of saxitoxin in the western basin, and we recommend that future Lake Erie cyanobacteria research should not solely focus on microcystins and planktonic blooms.

## 1. Introduction

Due to excessive nutrient loading and a warming climate, cyanobacterial harmful algal blooms (cHABs) events are a global issue that often leads to hypoxic zones, cyanotoxin production, disrupted food webs, and negative impacts on local economies (O’Neil et al., 2012; Paerl et al., 2016a). Lake Erie, the shallowest and most productive of the Laurentian Great Lakes, is widely known for significant cHABs, especially in the western basin (Michalak et al., 2013; Rinta-Kanto et al., 2009; Steffen et al., 2017). While microcystin (MC)-producing *Microcystis* often dominates the cyanobacterial community in Lake Erie’s western basin blooms, other toxin-producing cyanobacteria such as *Dolichospermum, Pseudanabaena, Planktothrix*, and *Aphanizomenon* have been detected (Berry et al., 2017; Christensen and Khan, 2020; Jankowiak et al., 2019). Although MCs are considered the most widespread cyanotoxin globally and in Lake Erie (Harke et al., 2016), saxitoxins (STXs) are becoming increasingly observed and can be produced by other cyanobacteria known to be found in freshwater systems (Christensen and Khan, 2020; Li et al., 2016; Loftin et al., 2016). Saxitoxins, which include saxitoxin and 57 documented STXs analogs (also referred to as paralytic shellfish toxins), are potent neurotoxins and found in both marine and freshwater systems (Llewellyn et al., 2001; Wiese et al., 2010). In Ohio, one of the biosynthetic genes responsible for STX production, called *sxtA*, has been reported in the offshore waters of Lake Erie’s central basin (Chaffin et al., 2019), the Maumee River (Laiveling et al., 2022), and in the Ohio EPA’s public water systems database (https://epa.ohio.gov/divisions-and-offices/drinking-and-ground-waters/public-water-systems/harmful-algal-blooms). These reports of *sxtA* imply that the management’s focus on MC production and concentration may be an oversight. Helping to understand which waters are prone to STX production would aid managers in protecting human health.

While planktonic *Microcystis* blooms have received much of the research and management focus in Lake Erie, benthic cyanobacterial blooms have also been documented (Bridgeman and Penamon, 2010). The filamentous benthic cyanobacterium *Microseira wollei* (formerly *Lyngbya wollei*) is common in Lake Erie (Bridgeman and Penamon, 2010) and known to produce multiple STXs (Smith et al., 2019)*, b*ut, there are many other benthic cyanobacteria taxa capable of producing STXs (Pokrzywinski et al., 2021; Wood et al., 2020). Research on benthic primary production has received much less attention compared to planktonic (Burford et al., 2020; Cantonati and Lowe, 2014). It is well known that light intensity, substrate type, and physical forces (currents, waves) are major factors constraining benthic algal biomass (Vadeboncoeur et al., 2008, 2006). Overall, there is a lack of understanding of the benthic cyanobacteria and their STX production potential in Lake Erie.

The overall objective of our study was to understand the prevalence of *sxtA* and STX in the planktonic and benthic habitats of western Lake Erie. Our specific objectives were to: 1) determine the spatial and temporal distribution of *sxtA* and STX in the water column, 2) determine how enrichments of phosphorus (P) and three forms of nitrogen (N) (nitrate, ammonium, and urea) affect *sxtA* and STX production in the water column, and 3) investigate how seasonality, depth, and nutrients affect the colonization and growth of STX-producing benthic cyanobacteria at one study location on Lake Erie at Put-in-Bay, Ohio. We approached these objectives across five bloom seasons (May to October during 2018–2022) that involved routine water sample collection and two separate *in-situ* experimental designs, which all took place congruently across two bloom years (May to October during 2018 and 2019). We use the term “saxitoxin” (STX) in this paper to reference STX (Ballot et al., 2016; Llewellyn et al., 2001) instead of paralytic shellfish toxins (PSTs) because saxitoxin is the parent molecule of PSTs, and the ELISA method has high cross-reactivity only with STX and the cross-reactivity with other STX congeners and other PSTs (guanotoxins and lyngbyatoxins) ranges from less than 0.2 % to 29 % (Gold Standard Diagnostics, 2022).

## 2. Methods and materials

### 2.1. Water column sample collection and analysis

We utilized three data sources to determine the spatial and temporal distribution of *sxtA* in the water column of Lake Erie’s western basin. First, we collected water samples in the open water of Lake Erie at three long-term monitoring locations during 2018 and 2019. Secondly, we accessed the Ohio EPA’s *sxtA* dataset for public water systems (PWS) in the western basin. Thirdly, we utilized the HABs Grab sample set to give a high spatial resolution across the entire western basin. Each data set is described in detail below.

**Fig. 1.**
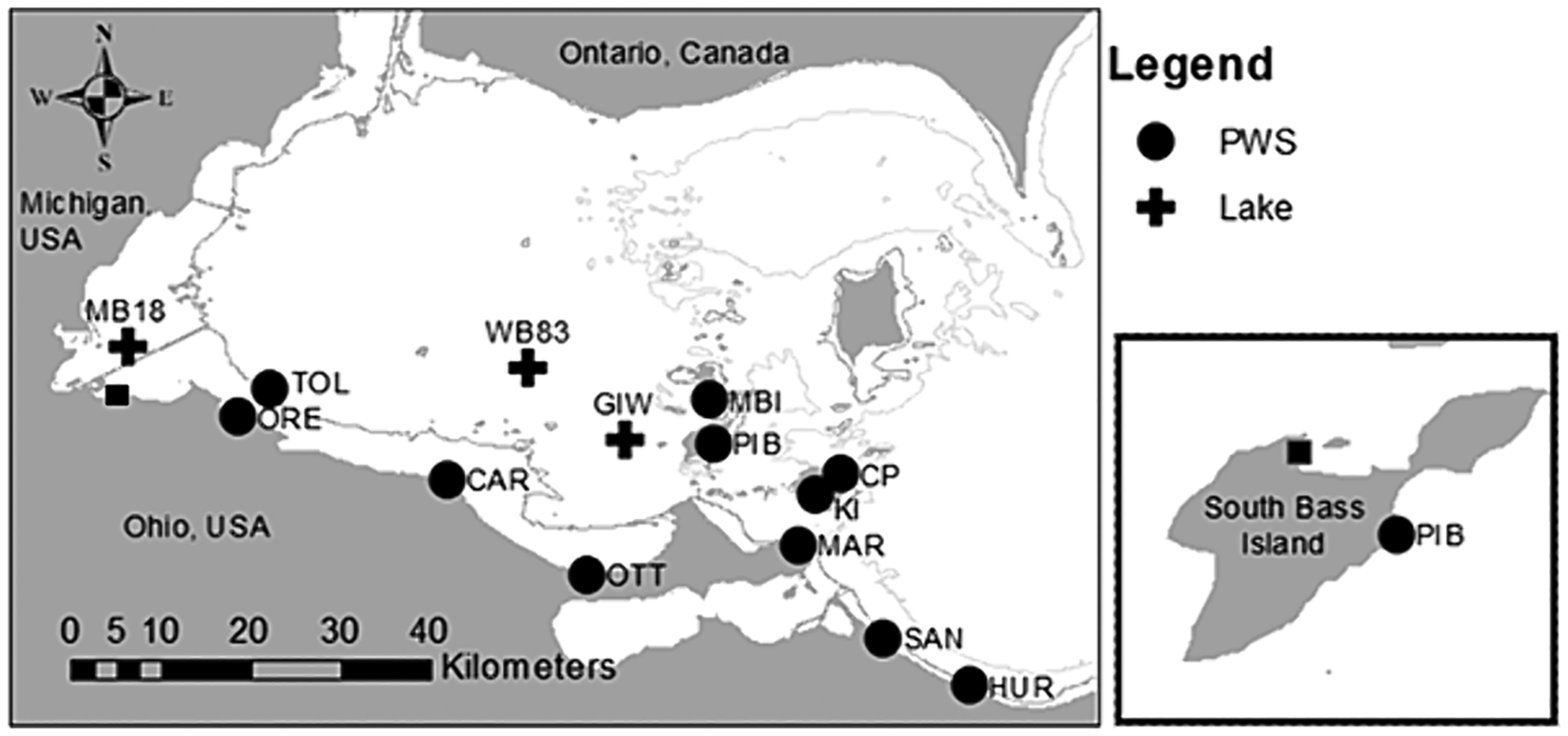
Location of grab samples collected in Lake Erie during 2018 and 2019 (crosses), public water systems source water intake (circles), and incubation experiment (squares). The incubation location at Put-in-Bay on South Bass Island was used for both the nutrient diffusing substrate and the WB83 water column experiments. The contour lines are 5 m and 10 m depth. See Table 1 for intake abbreviations full name.

For the first dataset at three open water sites (MB18, WB83, GIW; Fig. 1), we collected data throughout the summers of 2018 and 2019 at weekly to biweekly intervals. Upon the research vessel arrival at the station, the anchor was deployed, and we recorded water temperature, pH, turbidity (FTU), and Specific Conductivity (μS/cm) with a YSI EXO2 sonde. We collected grab samples with integrated tube samplers (0–2 m at MB18, 0–8 m at WB83 and GIW; Fig. 1) (Golnick et al., 2016). Sample water from the sampler was deposited into a clean 20-L bucket and then poured into 250-mL polyethylene terephthalate glycol (PETG) bottle (for total nutrient concentration), a 500-mL PETG bottle (for DNA), and a dark 2-L polycarbonate bottle for chlorophyll *a*. We also filtered ~ 50 mL of water with a 0.45 μm filter into a 60-mL PETG bottle for analysis of dissolved nutrients. All bottles were stored in a dark ice chest while transported back to the laboratory. Upon arriving at the laboratory (between 2 and 4 h after sample collection), we filtered samples for DNA and chlorophyll and placed the nutrient samples in a freezer at −20 °C.

For measurements of the *sxtA* gene, we filtered the sample (0.025–0.5 L) onto a Versapor acrylic copolymer 1.2 μm pore, 25 mm diameter disk filters, following the procedures in Laiveling et al. (2022). All samples were held at −80 °C until after the 2019 field season. We extracted DNA and quantified *sxtA* gene copies with the Phytoxigene^™^ CyanoDTec qPCR kits (https://www.phytoxigene.com/products, as listed in Laiveling et al. (2022). The assay limit of quantification is 25 copies/reaction at a 95 % CI. The Phytoxigene^™^ CyanoDTec qPCR kits are multiplexed for simultaneous quantification of the *sxtA*, *mcyE*, and *cyrA* genes, but we focused on *sxtA* for this study. Every sample was analyzed in duplicate.

Total P (TP) and total Kjeldahl N (TKN) concentrations were measured in the unfiltered water stored in the 250-mL bottle. Concentrations of nitrate, nitrite, ammonium, and dissolved reactive P (DRP) were measured on the filtered water stored in the 60-mL bottle. Total N concentration was calculated at the sum of nitrate, nitrite, and TKN. All analyses were conducted on a SEAL Analytical QuAAtro (Mequon, WI) segmented flow auto-analyzer following standard methods (Chaffin et al., 2021).

Plankton (50 to 500 mL, depending on biomass) was captured on Whatman^™^ GFF glass fiber filter (GF/F; 0.7 μm nominal pore size) to analyze chlorophyll *a* concentration. The filters were stored on silica gel in mini-Petri dishes at −80 °C until analysis. We followed the dimethyl sulfoxide extraction and quantification method (Golnick et al., 2016). We also used a FluoroProbe (bbe Moldaenke, GmbH) with a benchtop cuvette reader to determine chlorophyll *a* associated with cyanobacteria. The filter-extracted chlorophyll *a* concentration was used to normalize the FluoroProbe (Chaffin et al., 2021). FluoroProbe measurements were recorded upon returning to the laboratory and after the samples reached room temperature. Finally, we preserved a 100 mL sample with 1 % formalin for planktonic cyanobacteria identification and quantification. We used a FlowCam (model 8410, Yokogawa Fluid Imaging Technologies) to quantify planktonic cyanobacteria in the formalin-preserved sample in auto-image mode at 100X magnification. Image collection stopped after 8000 individual particles were imaged. Images were classified using a library in Visual Spreadsheet (#5.7.19) and then manually checked and reclassified as needed. The biovolume shape for each taxon was used following that of Hrycik et al. (2019). The particle average biovolume (μm^3^/particle) was then multiplied by particles/mL to calculate biovolume per mL (μm^3^/mL).

We accessed the Ohio EPA’s *sxtA* dataset for PWSs located in Lake Erie’s Western Basin for our second data source (https://epa.ohio.gov/divisions-and-offices/drinking-and-ground-waters/public-water-systems/harmful-algal-blooms). The PWS are required to collect samples (minimum 200 mL) biweekly from their raw water source tap to screen for total cyanobacteria (16S) and three cyanotoxin genes (*sxtA*, *mcyE*, and *cyrA*). The PWS water samples were chilled on ice and transported to an Ohio EPA-certified laboratory, where they were processed and analyzed by the Ohio EPA DES qPCR Multi-Plex Molecular Method 705.0 Version 1 (Ohio E.P.A., 2018). Gene copies are reported to Ohio EPA. The reporting limit is 180 gene copies per mL. We downloaded PWS data from the Ohio EPA database for years 2019–2022. We omitted 2018 data due to an error in the Phytoxigene kit in which the *sxtA* fluorescence probe was incorrectly linked to the *cyrA* gene, which resulted in all non-detects during 2018 (unpublished data).

The third water column sample set was from the HABs Grabs conducted on 9 August 2018 and 7 August 2019 (Chaffin et al., 2021). The HABs Grab sample events gave a high spatial resolution snapshot of cyanobacteria in the western basin. One hundred samples were collected in U.S.A designated waters in 2018, and 172 samples were collected in the entire basin in 2019. All samples were collected with a two-meter-long tube sampler, the water was quickly deposited into a clean 20-L bucket, and then poured into 2.4L PETG bottles (Chaffin et al., 2021). The water samples were held in an ice chest and transported back to the laboratory. Once at the laboratory, sample filtering began immediately following the same methods listed above. The *sxtA* gene was quantified with the Phytoxigene kits, following the methods stated above. We also tested for STX in 39 selected samples from the 2019 HABs Grab with a range of *sxtA* gene copies. Water (20 mL) from the PETG bottle was poured into 60-mL amber glass vials and placed in the freezer, then the samples were subjected to three freeze–thaw cycles to lyse the cells (Ohio EPA, 2016). After the third thaw, the cellular debris was removed from a 2-mL subsample by filtration with a GMF (0.45 μm) syringe filter and deposited into a 4-mL glass vial, and then the Eurofin Abraxis (now named Gold Standard Diagnostics) STX preservative (#53001L) was added. We used Eurofins Abraxis enzyme-linked immunosorbent assay (ELISA) kits to quantify STX (#52255B) on the Eurofins Abraxis automated ELISA instrument CAAS Cube (#475006).

### 2.2. Water column experiments

We determined the effects of P and N enrichment on *sxtA* and STX production with water collected from sites MB18 and WB83. Initially, the purpose of these experiments was to quantify microcystin and *mcyE* production in the western basin (Chaffin et al., 2022), but because the Phytoxigene kits are multiplexed (Al-Tebrineh et al., 2012), we analyzed the *sxtA* data for this project. The complete experimental design can be found in Chaffin et al. (2022). Briefly, we collected surface water (40 L) twice a month from sites MB18 and WB83. The lake water was poured into 12 2.4-L clear PETG bottles. We had four treatments: 1) a control without enrichment, 2) 1 μmol/L P (as KH_2_PO_4_) and 100 μmol/L nitrate (NaNO_3_) enrichment, 3) 1 μmol/L P and 100 μmol/L ammonium (NH_4_Cl), and 4) 1 μmol/L P and 100 μmol/L urea-N (50 μmol/L urea). Each treatment was replicated with three separate bottles. Samples were collected for *sxtA* and particulate STX (captured on a filter) before and after 72 h of incubation suspended from docks *in situ* in Lake Erie. The MB18 experiments were incubated in Maumee Bay at the University of Toledo’s Lake Erie Center, whereas the WB83 experiments were incubated in Put-in-Bay at the Ohio State University’s Stone Lab (Fig. 1). Cyanobacteria-specific chl *a* measured with a bbe FluoroProbe was used as a metric of cyanobacterial biomass (Chaffin et al., 2022).

### 2.3. Benthic cyanobacteria

We deployed nutrient diffusing substrata (NDS) to determine the impact of nutrients, depth, and season on the colonization and growth of potential-STX-producing benthic cyanobacteria. NDS are artificial substrates deployed *in situ* that leach nutrients through a porous substrate to determine if benthic algae are nutrient-limited (Tank et al., 2017). We used 30-mL polycon cups for each NDS replicate (Capps et al., 2011; Tank et al., 2017). We drilled a 19 mm diameter hole (2.85 cm^2^) into each lid and then washed the cups in phosphate-free soap. The cups were filled with autoclaved 2 % agar containing one of the six nutrient treatments: 1) a control without enrichment, 2) P-only enrichment (0.5 mmol/L as KH_2_PO_4_), 3) nitrate-only (10 mmol/L as NaNO_3_), 4) ammonium-only (10 mmol/L as NH_4_Cl), 5) P and nitrate, and 6) P and ammonium. A total of 20 NDS replicate cups were made for each nutrient treatment, providing 10 cups to be used at each depth (0.5 m and 2.0 m from the surface). As the agar cooled and solidified, each cup was topped with a pre-combusted fritted glass disk (EA Consumables, Porous Crucible Cover PN: 528–042) to ensure that the disk was exposed on the top of the agar. The cups were closed and reinforced with gorilla tape to keep the lid closed throughout the deployment. The cups were secured to a PVC L-bar (US Plastics #45031) in random order with zip ties and monofilament fishing line. Next, the L-bars with NDS cups were secured in one of two plastic crates (one for each depth) (61 cm × 41 cm × 13 cm) with zip ties. The NDS crates were then deployed in Put-in-Bay, Lake Erie, at Stone Laboratory (Fig. 1) suspended off a dock at 0.5 m and 2.0 m from the water surface with rope and stabilized with concrete blocks (Electronic Supplementary Material (ESM) Fig. S1). The NDS were deployed for 14 days. Fresh agar and new cups were prepared before each deployment.

At the end of each experiment, the NDS were removed from the lake and brought into the laboratory. All NDS replicates were immediately analyzed for the abundance of green algae, diatoms, and cyanobacteria with a FluoroProbe equipped with a BenthoFluor attachment (bbe Moldaenke, Germany). Because the BenthoFluor attachment is an optic fiber that directly transmits the FluoroProbe signals, the units from the FluoroProbe (μg chl *a*/L) were considered relative units for this study. The NDS were kept wet with lake water while analysis occurred. After the BenthoFluor evaluation, the frits were removed from the NDS. Three frits from each treatment were placed in a 50-mL Falcon tube with 10 mL of deionized water and stored at −20 °C for STX analysis. Another three frits were stored in 50-mL Falcon tubes without deionized water and stored at −80 °C for DNA extraction. Three more frits were dried and weighed for analysis of organic matter. Finally, the tenth frit was stored in a 125-mL plastic bottle with 10 mL of 1 % formalin for algae identification by microscopy using the keys of (Wehr et al., 2014) with new taxonomic updates (Hašler et al., 2012; Meriluoto et al., 2016; Strunecký et al., 2014, 2013).

The NDS saxitoxin samples were analyzed similarly to the water column samples. First, the NDS samples for STX analysis were frozen/thawed three times, and then the cellular debris was removed from a 2-mL subsample by filtration with a GMF (0.45 μm) syringe filter and deposited into a 4-mL glass vial. STX concentration was measured in the lysate with the Eurofins Abraxis ELISA kits, and then STX was back-calculated to ng/cm^2^ by multiplying the ELISA result by 10 mL (volume of deionized water) and dividing by 2.85 cm^2^ (the area of the frit).

We had planned to quantify the *sxtA* gene but were unsuccessful in extracting DNA due to method limitations. The limitations included potential DNA binding due the glass nature of the frits, the size of frits, and Covid-19 supply chain constraints. We omitted the organic matter data because microinvertebrate grazers were observed by microscopy, and we could not differentiate alive organic matter from organic debris that settled onto the NDS from the water column.

### 2.4. Data analysis

Using the Kriging function, we conducted spatial interpolations of the *sxtA* gene copies and cyanobacterial-chl a collected during the HABs Grabs with ArcGIS v10.3. The Kriging output maps were cropped to the area sampled.

We conducted a correlation analysis between the *sxtA* gene copy data and the environmental data collected from sites MB18, GIW, and WB-83. We analyzed the *sxtA* gene copy data from the water column nutrient enrichment bioassays with an analysis of variance (ANOVA) and a post-hoc Tukey test to determine differences among treatments. To analyze the NDS data, a 3-factor ANOVA was used to determine the difference in SXT and cyanobacteria biomass among nutrient treatment, depth level, and season. IBM SPSS version 27 was used for all ANOVAs.

## 3. Results

### 3.1. sxtA in the water column

**Fig. 2.**
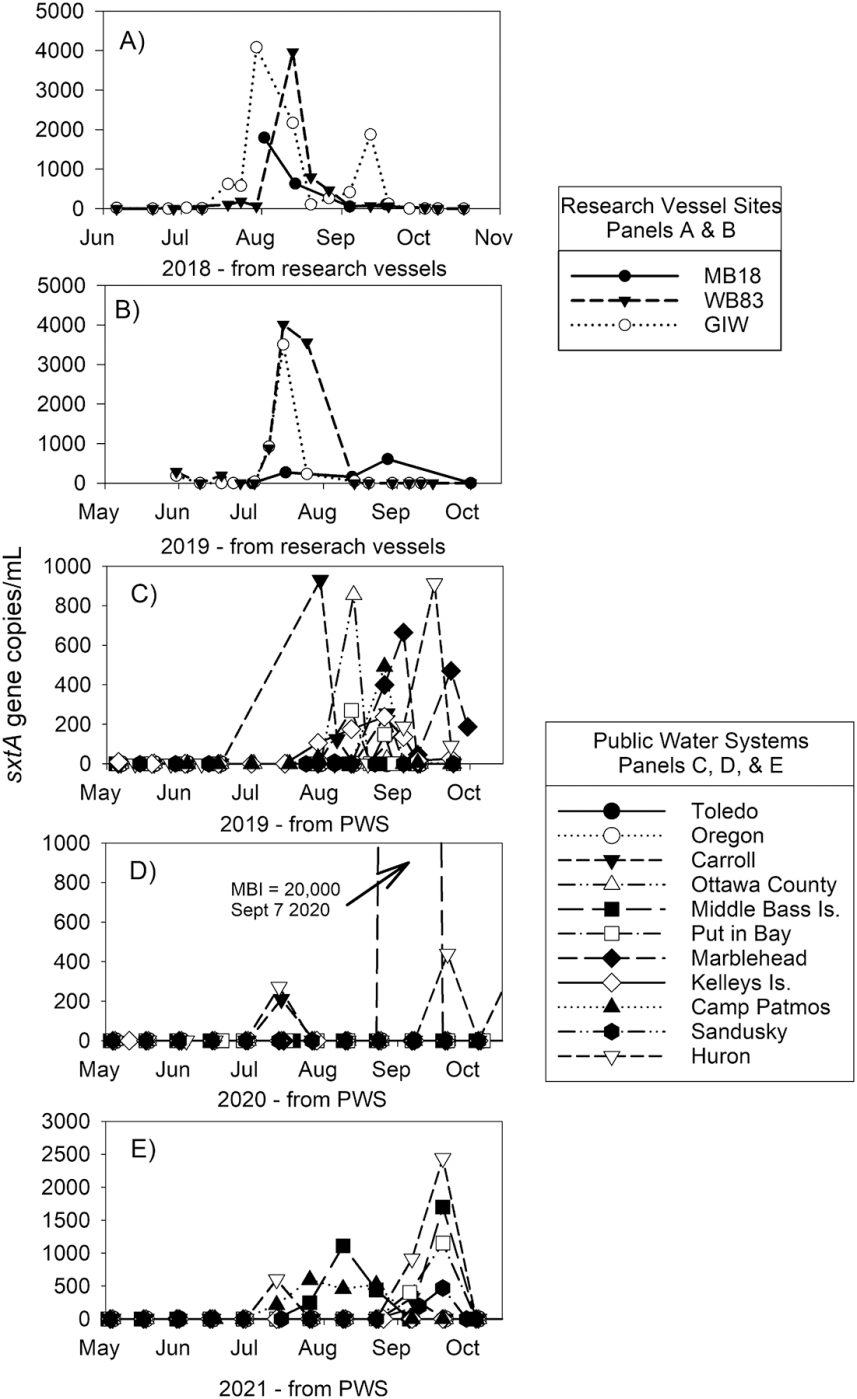
*sxtA* gene copies (/mL) in the western basin during 2018–2021. Panels A and B were collected aboard research vessels during 2018 (A) and 2019 (B), whereas panels C, D, and E show *sxtA* data collected from the intakes of 11 public water systems (PWS) in 2019, 2020, and 2021. The PWS intakes in the legend are arranged west to east. Note the difference in the Y-axis scale. 2022 is not shown in the figure due to low number of non-detects (Table 1).

Concentrations of *sxtA* in the open water western basin samples ranged from below detection to 4,083 gc/mL (at site GIW on 30 July 2018) (Fig. 2). Sites GIW and WB83 had between 2 and 10 times higher *sxtA* gene copies than site MB18 on each sampled date. In 2018, site GIW had two *sxtA* peaks in late July (4,083 gc/mL) and mid-September (1,880 gc/mL), whereas MB18 and WB83 had one peak in late July and early August, 1,799 gc/mL and 3,964 gc/mL, respectively (Fig. 2A). In 2019, GIW and WB83 each had one *sxtA* peak in mid-July (3,508 – 4,014 gc/mL), whereas *sxtA* at MB18 remained low throughout the year (<610 gc/mL; Fig. 2B). There was a weak but significant correlation between *sxtA* gene copies and water temperature (p = 0.027; r = 0.263) and no other variables including cyanobacterial biovolumes correlated with *sxtA* (ESM Table S1). *Microcystis* was the most abundant cyanobacterial genus throughout both 2018 and 2019 (ESM Fig. S3). Genera known to have potential STX-production, *Aphanizomenon*, *Dolichospermum*, and *Planktothrix*, were observed during 2018 and 2019 (ESM Fig. S4), but there was no apparent temporal or spatial pattern among them.

The two most western PWS (Toledo and Oregon) did not have *sxtA* detections between 2019 and 2022 (Table 1). Across all years, Camp Patmos (on Kelley’s Island) and Huron PWSs, which are on the eastern edge of the western basin, had the most frequent *sxtA* detections (26.5 % and 18.4 %, respectively, Table 1). Across all PWSs, 2019 had the most frequent *sxtA* detections (22.8 %) and 2022 the lowest *sxtA* detections (3.2 %). The temporal pattern of *sxtA* gene copies at the PWS was similar to the open water sites with ephemeral peaks followed by declines to low or non-detect levels. During 2019, the timing of *sxtA* peak at each PWS followed a general pattern of occurring earliest in the most western PWS with later detections in the east (Fig. 2C). In 2019, Carroll PWS (the westernmost PWS) *sxtA* peak occurred on 30 July. The *sxtA* peak moved west to east, reaching Ottawa County PWS on 13 August, Marblehead PWS on 3 September, and finally Huron PWS, the easternmost PWS in the western basin, on 16 September (Fig. 2 C).

### 3.2. HABs Grab

**Fig. 3.**
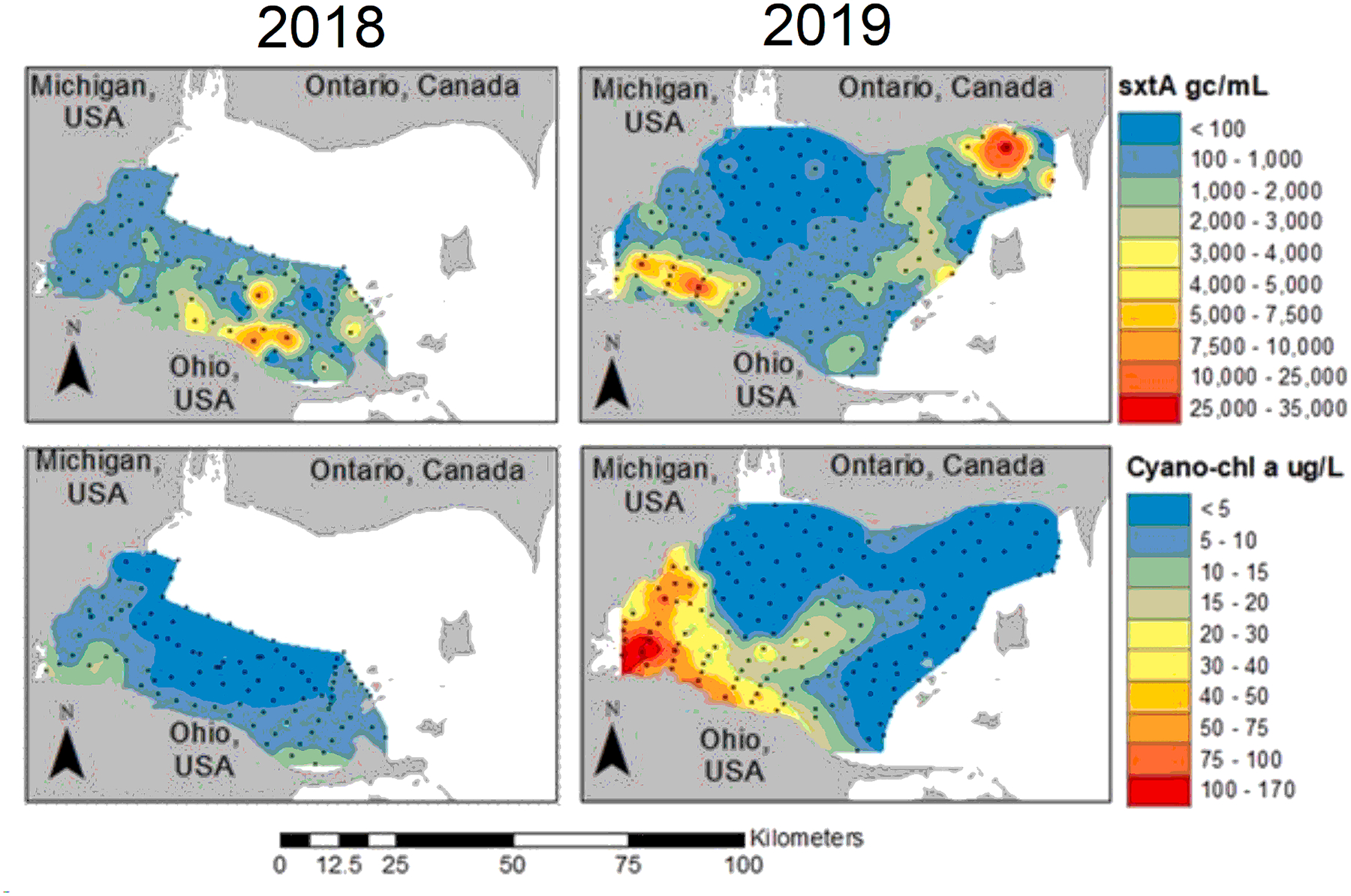
*sxtA* gene copies (/mL, top row) and cyanobacteria-specific chlorophyll *a* concentration (μg/L, bottom row) collected on the two HABs Grab events on August 9, 2018 (left column) and August 7, 2019 (right column). The black dots indicate sample location collection.

The HABs Grab data showed *sxtA* throughout the western basin during early August of 2018 and 2019 (Fig. 3). On 9 August 2018, 91 of the 100 samples had quantifiable *sxtA*, while 109 of the 171 samples collected on 7 August 2019 had quantifiable *sxtA*. In 2019, there was no *sxtA* detected in the area impacted by the Detroit River outflow (northwest area of the basin), which corresponded with cyanobacteria-chl *a* less than 5 μg/L. The northwest area of the basin was not sampled during the 2018 HABs Grab, so a basin-wide average could not be calculated without basis. Hotspots of *sxtA* concentration varied by location year to year and did not correspond with the cyanobacteria-chl *a* concentration peaks. The highest 2018 *sxtA* concentration (10,482 gc/mL) was measured in the vicinity of the Carroll PWS (Fig. 1). Comparatively, in 2019, the *sxtA* detection hotspots were located around both the Maumee Bay (southwest of the basin) and Pigeon Bay (41,536 gc/mL, northeast of the basin in Canadian waters) regions. The HABs Grab *sxtA* detections did not correlate with any other measured parameter (p > 0.1, r < 0.14; ESM Table S1). Saxitoxins were not detected in any HABs Grab samples.

### 3.3. In-situ Lake Experiments

#### 3.3.1. Nutrient Addition Bottle Experiments

**Fig. 4.**
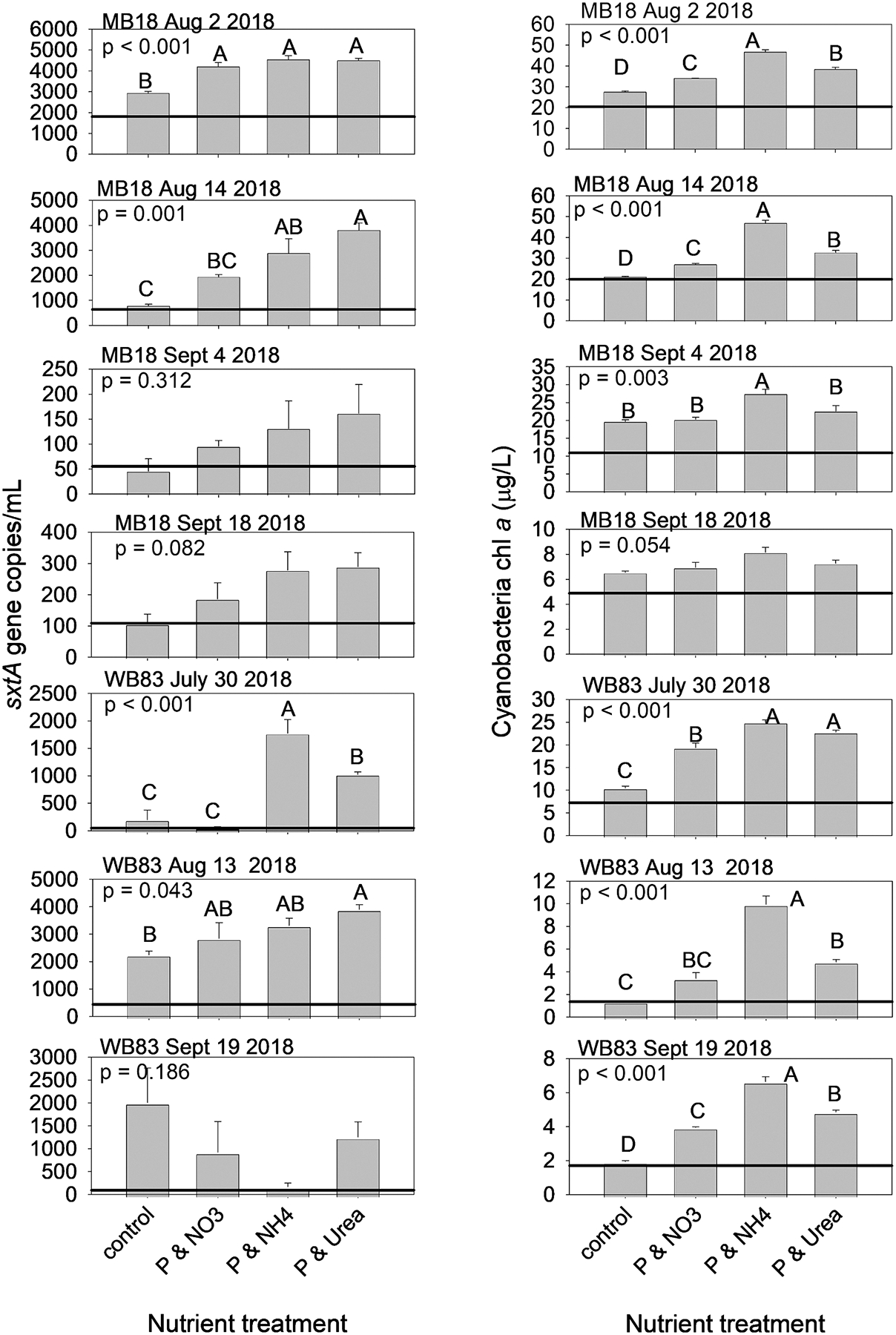
*sxtA* gene copies (left column) and cyanobacteria-specific chlorophyll concentration (right column) in nutrient enrichment bioassay experiments conducted during 2018 with water collected from site MB18 in Maumee Bay (top 4 rows) and WB83 in the middle of the western basin (bottom 3 rows). Only shown are the experiments in which *sxtA* was detected. The solid horizontal line are the initial concentrations. Note the difference in Y-axis scale. Letters above the bars indicate Tukey test grouping with the mean of A > mean of B > mean of C.

**Fig. 5.**
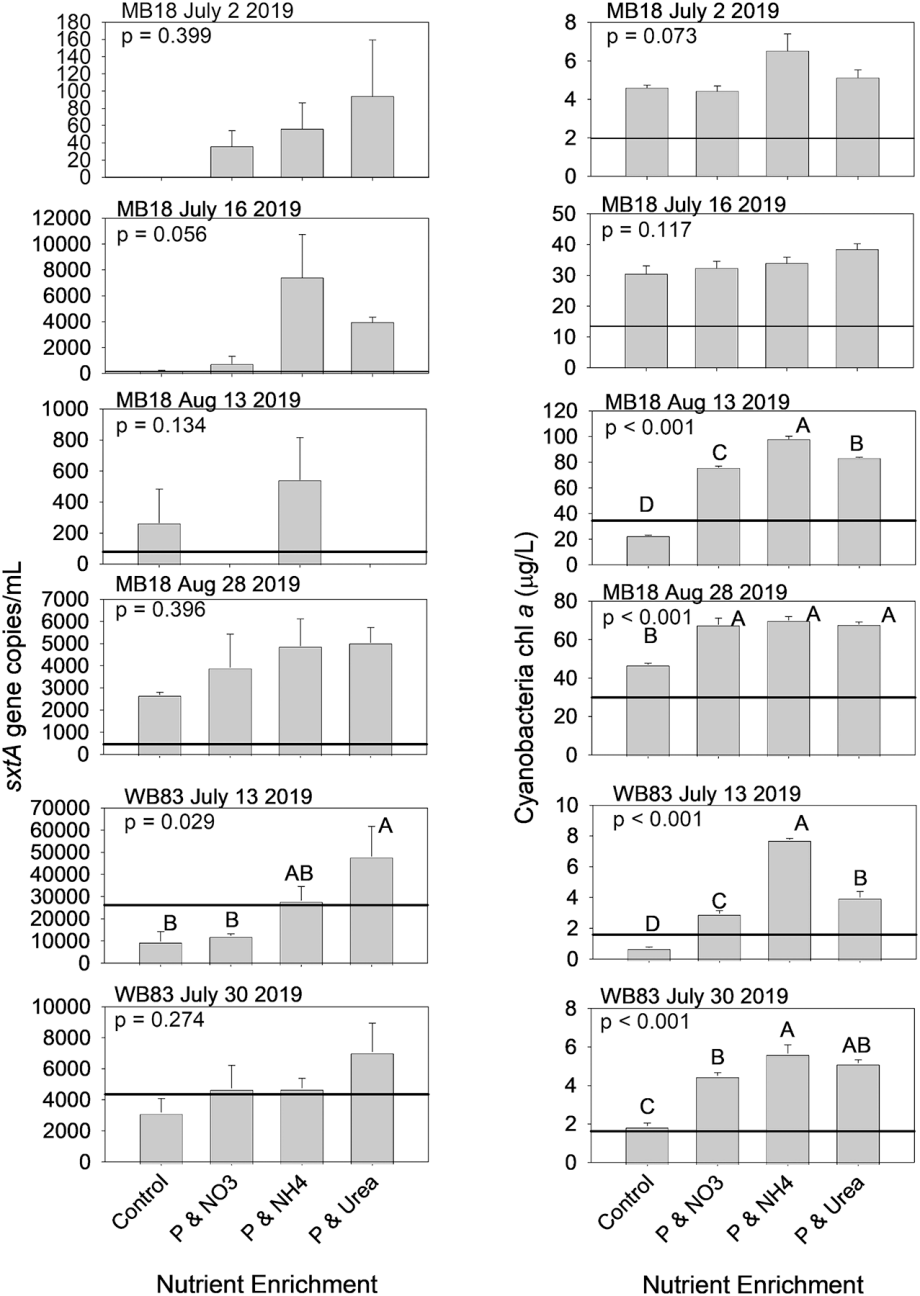
*sxtA* gene copies (left column) and cyanobacteria-specific chlorophyll concentration (right column) in nutrient enrichment bioassay experiments conducted during 2019 with water collected from site MB18 in Maumee Bay (top 4 rows) and WB83 in the middle of the western basin (bottom 2 rows). Only shown are the experiments in which *sxtA* was detected. The solid horizontal line are the initial concentrations. Note the difference in Y-axis scale. Letters above the bars indicate Tukey test grouping with the mean of A > mean of B > mean of C.

During 2018, seven nutrient addition bottle experiments had detectable *sxtA*, and four of the seven had significant differences among treatments (p < 0.05; Fig. 4), but a fifth experiment was nearly significant (p = 0.082). In 2019, six experiments had detectable *sxtA*, and only one of six had significant differences among treatments (Fig. 5), but another experiment was nearly significant (p = 0.056). Among the experiments with significant differences among treatments (both years, both sites), the P and N enrichment treatments resulted in higher levels of *sxtA* than the initial level and in the control. The P and ammonium and P and urea enrichments resulted in the highest *sxtA*. Whereas the *sxtA* response to P and nitrate enrichment ranged from not significant from the control (ex: WB83 30 July 2018; WB83 13 July 2019), significantly greater than the control but significantly lower than P and ammonium (ex: MB18 14 August 2018), and significantly greater than the control and not significant from the P and ammonium and urea enrichments (ex: MB18 2 August 2018).

Cyanobacteria-specific chl *a* concentration was significantly different among treatments in 10 of the 13 experiments (p < 0.05; Figs. 4 and 5), and one experiment was nearly significant (MB18 18 September 2018, p = 0.054). In the two experiments without significant differences (MB18 2 July 2019, and 16 July 2019), chl *a* in the controls were more than double that of the initial concentration, indicating the high concentrations of ambient DRP and nitrate (ESM Figure S2) alleviated short-term nutrient limitation. In the 10 experiments with significant differences in chl *a* concentrations among treatments, the enrichments of P and ammonium resulted in the highest chl *a* concentrations, the P and urea and the P and nitrate resulted in intermediate chl *a* concentrations, and the control had the lowest chl *a* concentrations.

There were five experiments (across both sites and years) in which P and N enrichment, regardless of N form, resulted in higher concentrations of both *sxtA* and cyanobacteria chl *a* (ex: MB18 2 August 2018, Fig. 4; WB83 13 July 2019, Fig. 5). There were six experiments that increased cyanobacteria chl *a* with P and N enrichment but *sxtA* did not (ex: MB18 28 August 2019, Fig. 5). There were two experiments in which both *sxtA* and cyanobacteria chl *a* did not differ among control and nutrient enrichment (MB18 2 July 2019, and 16 July 2019). There were no experiments in which *sxtA* responded to nutrient enrichments while cyanobacteria chl *a* did not.

Saxitoxin was not detected in any bottle experiment. This could be due to the cyanobacteria did not produce STX, cyanobacteria produced a STX congener not detected by ELISA, or due to the method of measuring particulate STX (extracted from a filter) when STX tends to be extra-cellular (Jørgensen et al., 2022).

#### 3.3.2. Nutrient Diffusing Substrata Experiments

**Fig. 6.**
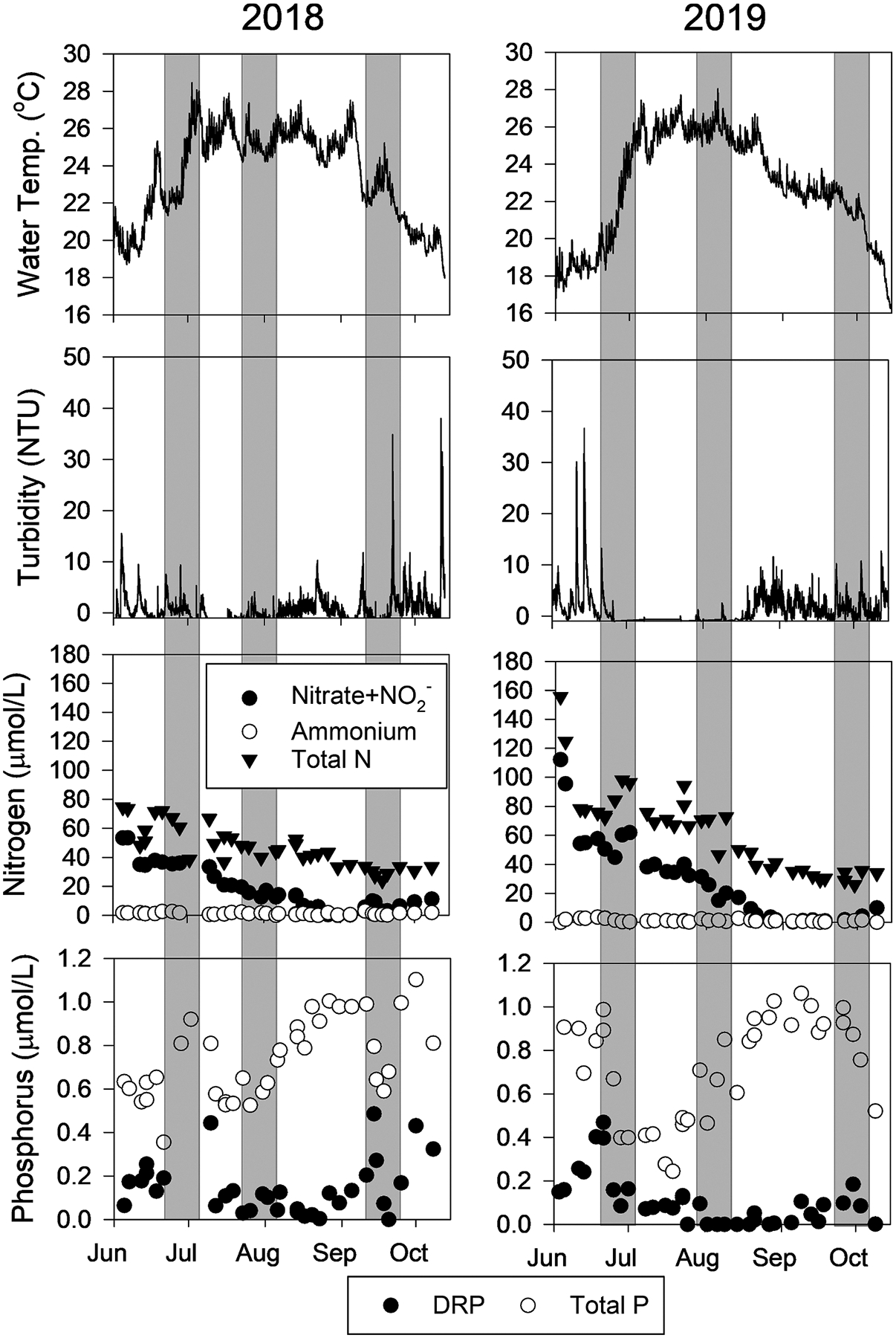
Ambient environmental data during the summers of 2018 and 2019 at Put-in-Bay, Lake Erie. The gray boxes indicate the timing of the six nutrient-diffusing substrata experiments.

We deployed NDS experiments in late June, August, and late September in both 2018 and 2019, and within each time frame, the ambient water conditions were similar between both years (Fig. 6). Both June NDS experiments were characterized by rapidly warming water (22 °C to 27 °C in 2018, 19 °C to 26 °C in 2019), mild turbidity (0–10 NTUs), high nitrate concentrations (>30 μmol/L), and detectable DRP. The August NDS experiments were characterized by warm and stable water temperature (25–26 °C), the clearest water (NTU < 3), declining nitrate concentrations (10–20 μmol/L), and the lowest TP (0.4 – 0.6 μmol/L) and DRP (<0.05 μmol/L) concentrations. The September NDS experiments were characterized by cooler water temperature than August (20–24 °C), unstable turbidity (0—35 NTU), low nitrate concentrations (<10 μmol/L), and the highest TP and DRP concentrations of the year (0.6 – 1.0 μmol/L, 0.1 – 0.5 μmol/L, respectively).

**Fig. 7.**
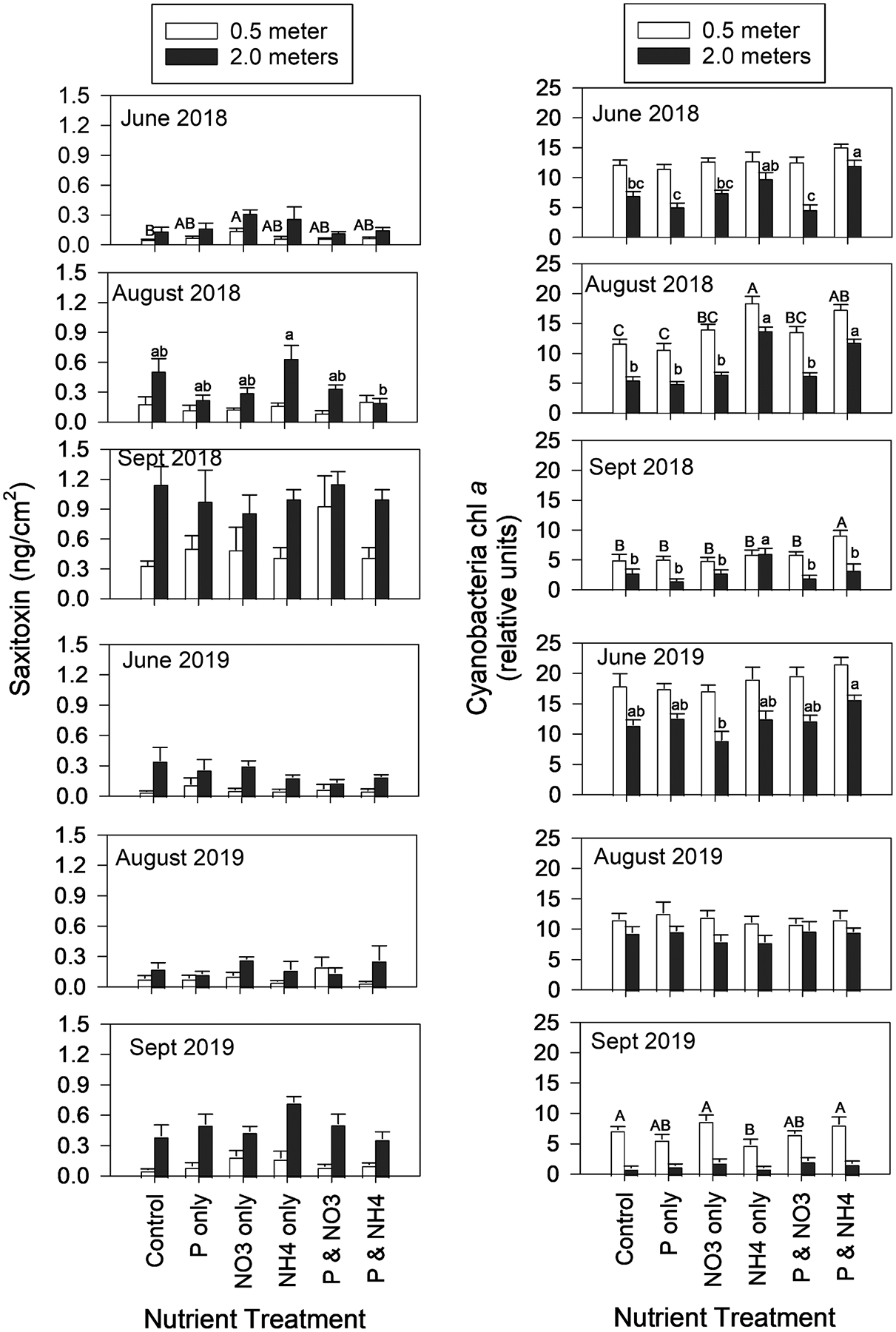
Saxitoxin (left column, ng/cm^2^) and cyanobacteria-specific chlorophyll *a* (right column, relative units) on six nutrient-diffusing substrata experiments after 14 days of incubation in Put-in-Bay, Lake Erie, during 2018 and 2019. Letters above the bars indicate Tukey test grouping with the mean of A > mean of B > mean of C on the main effects of nutrients within each depth level and experiment. Capitalized letters are for the 0.5 m depth, and lowercase letters are for the 2.0 m depth. The lack of letters above bars indicates the nutrients main effect for that experiment and depth was not significant (p > 0.05).

Saxitoxin and cyanobacterial-chl *a* concentrations followed different patterns in the NDS experiments (Fig. 7; Table 2). In general, STX ng/cm^2^ was lowest in June (of both years) and highest in September, while cyanobacteria-chl *a* was highest in June and lowest in September. Saxitoxin was higher at the 2.0-meter depth than the 0.5-meter depth, while cyanobacteria-chl *a* was higher at 0.5 m. Saxitoxin was unaffected by nutrient enrichment (p > 0.5, Table 2), while cyanobacteria-chl *a* was significantly different among nutrient enrichments (p < 0.05, Table 2). Saxitoxin was significantly affected by the interaction between depth and season, and depth and season were each significant on their own (Table 2). Cyanobacterial-chl *a* concentrations of the NDS were affected by the interactions between season*depth, season*treatment, and depth*treatment (the 3-factor interaction was not significant), and each factor was significant on its own (Table 2). The interaction among factors complicated the interpretation of the nutrient enrichment cyanobacteria-chl *a*, but there are a few general patterns across all experiments. First, the cyanobacteria-chl *a* within each experiment and depth level did not significantly differ between the control and P-only enrichments. For example, in the June 2018 experiment, cyanobacterial-chl *a* in the 0.5-meter depth level were 12.13 ± 0.83 (mean ± 1 standard error; relative units – see methods section) and 11.42 ± 0.77 in the control and P-only enrichment respectively, and 6.92 ± 0.75 and 5.00 ± 0.73 in the control and P-only enrichment, respectively at the 2.0-meter depth level. Secondly, the differences among nutrient enrichment tended to be more pronounced at the deeper depth level than the shallow/higher light level (hence the interaction between the two factors in 2018, p = 0.001). Third, in five of the six experiments (sans August 2019), the ammonium-only or the P and ammonium enrichments resulted in the highest cyanobacterial-chl *a* within each depth level.

Between 13 and 25 genera of benthic algae colonized the NDS experiments (Table 3). Among the cyanobacteria, *Leptolyngbya* and *Pseudanabaena* were observed in all experiments except the July 2018 experiment. Nine other cyanobacterial genera, including coccoid and filamentous forms, were observed throughout our study. Eukaryotic algae observed in all experiments were *Pediastrum*, *Scenedesmus*, *Meloseria*, *Navicula*-like, and *Nitzschia*-like genera. Filamentous green algae (*Oedogonium*, *Stigeoclonium*, and *Spirogrya*) were observed in all experiments, but the genera differed among experiments. Notably, our study did not observe the typical nuisance benthic green alga *Cladophora*, and the filamentous cyanobacterium *Microseira* (“*Lyngbya*”) was only observed in one experiment. We could not quantify the benthic algae by counts present due to the inability to remove all algae from the glass frit substrate. Microinvertebrate grazers were also observed (i.e. Gastrotricha). Because benthic cyanobacteria taxonomy can be ambiguous, we included micrographs of example specimens in ESM Appendix S1.

## 4. Discussion

### 4.1. Spatiotemporal Distribution

This study used multiple datasets involving different monitoring events and experimental procedures to help understand the spatiotemporal dynamics of STX and *sxtA* in the western basin of Lake Erie. Every dataset included in this study found either *sxtA* or STX throughout multiple sites, habitats, sampling seasons, and years, especially 2018 and 2019. While the “HABs Grab” event was only a day-long snapshot, it provided basin-wide evaluation. The weekly to bi-weekly grab samples from research vessels and PWS provided a temporal dataset. Collectively, these datasets showed that most of the western basin has the potential for *sxtA* detection, except for the Detroit River-impacted area in the northwest area of the basin. Furthermore, these datasets show “hotspots” of *sxtA* that were ephemeral. The ephemeral nature of *sxtA* in the western basin is similar to *sxtA* found in the Maumee River (Laiveling et al., 2022) and the central basin of Lake Erie (Chaffin et al., 2019).

The lack of correlation between cyanobacterial taxa biovolume and *sxtA* did not provide any insights into which taxa were responsible for *sxtA*. *Dolichospermum* and *Aphanizomenon*, two genera that have been associated with *sxtA* elsewhere (Christensen and Khan, 2020), were observed in low biovolumes relative to *Microcystis* in the western basin throughout our study. However, a recent study by Yancey et al., (2023b) showed that one strain of *Dolichospermum* and one strain of *Aphanizomenon* collected from western Lake Erie during 2014 did not have the *sxtA* gene. In spite of this finding, there are likely multiple strains of both genera, as is with the case of *Microcystis* in western Lake Erie (Yancey et al., 2023a). The isolated hot spots of *sxtA* and the west-to-east sequential peaks of *sxtA* observed at the PWS during 2019 suggest that there may be cryptic strains of *Dolichospermum* and/or *Aphanizomenon* with *sxtA*. Genomic studies are needed to reveal which taxa have the *sxtA* and to determine if the gene is transcribed, which could elucidate bloom phenology and help determine the environmental factors that select the *sxtA* containing strain. While *Dolichospermum* and *Aphanizomenon* are often in low biovolumes compared to *Microcystis*, there have been times when *Dolichospermum* dominated the cyanobacterial community in the western basin. For example, the 2010 and 2011 cyanobacterial bloom switched from *Microcystis* to *Dolichospermum* in September following prolonged N-limitation (Chaffin et al., 2013), and the 2022 bloom switched to *Dolichospermum* in September which remained in high biomasses into mid-November (personal observation). Hence, continued monitoring of *sxtA* and further studies are needed.

In addition to finding *sxtA* in the water column, we found that the benthos can be a source of STX (although our methods prevented us from detecting *sxtA*). *Microseira wollei* (formally *Lyngbya wollei* (McGregor and Sendall, 2015)) is a common and highly abundant filamentous cyanobacterium that forms dense mats in wave-protected areas of western Lake Erie (Bridgeman et al., 2012; Bridgeman and Penamon, 2010). *Microseira wollei* in New York lakes was found to be a source of paralytic shellfish toxins (PSTS), but lyngbyatoxins, GTX-3, and GTX-5 made up the majority of the PSTs and STX and other PSTs were less than 3 % of all PSTs (Smith et al., 2019). In addition to *Microseira wollei*, many other cyanobacteria colonized the NDS experiments, and lyngbyatoxins may not be detected by the ELISA method (Smith et al., 2019) or have a low cross-reactivity of 13 % (Gold Standard Diagnostics, 2022). This suggests that other cyanobacteria could have been the source of STX in the NDS experiments. Many other benthic cyanobacteria are capable of cyanotoxin production, including STX (Catherine et al., 2013; Wood et al., 2020); however, STX is less screened for in benthic samples when compared to microcystins and anatoxins (Catherine et al., 2013; Wood et al., 2020).

The taxonomy of benthic filamentous cyanobacteria is rather ambiguous, which complicates the determination of potential toxin producers. Genera and species are being split while others are combined. For example, between 2014 and 2021, at least 273 species in 140 genera were described (Strunecký et al., 2023), and the morphological diversity within a taxon can be very high and influenced by environmental conditions (Strunecký et al., 2013). These make the identification of benthic cyanobacteria by microscopy challenging. One such example is *Phormidium*. *Phormidium* was reclassified as *Microcoleus* more than 10 years ago (Hašler et al., 2012; Strunecký et al., 2013), but more recent reports of *Phormidium* blooms continue (McAllister et al., 2016; Wood et al., 2020). A species of *Kamptonema* (*K. capsicum*) was moved from *Oscillatoria* to *Phormidium* and recently to *Kamptonema* (Vinogradova and Nuriyeva, 2020). The life stages of the filaments can also confuse taxonomists (Raabováet al., 2019). Many recent keys do not account for recent name changes. *Phormidium* is a known STX producer (McAllister et al., 2016; Wood et al., 2020), but it is unclear if the “*Phormidium*” we observed was a STX producer. Additionally, other filamentous genera we observed such as *Leptolyngbya* and *Kamptonema* have been reported to produce anatoxin-a and cylindrospermopsins, respectively (Shishido et al., 2023; Tavakoli et al., 2021). While our report does not identify the putative STX producer, we highlight that benthic cyanobacteria can be a source of cyanotoxins.

Lake Erie’s management strategy has primarily been focused on microcystins due to their common occurrence in the western basin. However, with technological advancements (such as the adaption of multiplex qPCR), there has been an increased awareness of STX in Lake Erie. Screening for *sxtA* with multiplexed qPCR is a good practice and may give insight into latent STX. The PWS data were collected at biweekly intervals, and peaks in *sxtA* were detected at this sampling frequency (Fig. 2). If *sxtA* exceeds 500 gc/mL at a PWS, that PWS will be required to increase sample frequency to weekly and a follow up STX (by ELISA) sample may be requested. Because most *sxtA* levels in the Maumee River (Laiveling et al., 2022), the western basin (this study), and the central basin (Chaffin et al., 2019) were low (<5000 gc/mL) compared to other locations in Ohio and elsewhere (Jørgensen et al., 2022; Kramer et al., 2018; Podduturi et al., 2021) and the correlation between *sxtA* and STX concentrations (Al-Tebrineh et al., 2010; Savela et al., 2015), this monitoring frequency is adequate for now to protect human health. However, based on the inconsistency of *sxtA* detections, biweekly monitoring may be too infrequent to determine potential drivers if conditions in the lake change and STX-producing cyanobacteria become more common.

### 4.2. Potential Drivers of STX and sxtA

Potential STX-producing cyanobacteria in the plankton (i.e., *sxtA* gc/mL) increased with P and N additions in 6 of the 13 experiments, but total cyanobacteria biomass increased with nutrients in 10 of the 13 experiments, suggesting the nutrient status differed among the potential STX-producers and the broader cyanobacterial community. Furthermore, there were nuanced responses to the three forms of N. Nutrient-limited growth among Lake Erie cyanobacteria (and all phytoplankton in general) was expected based on many previous studies (Barnard et al., 2021; Chaffin et al., 2013; Jankowiak et al., 2019). However, many other environmental factors that were not addressed in our study, such as light intensity, water temperature, and carbon dioxide, have been shown to impact STX production and growth of STX-producing cyanobacteria. Previous studies using culture-based approaches have provided mixed results (Pearson et al., 2016). To use temperature as an example, one experiment (Casero et al., 2014) showed that *Aphanizomenon* had the highest STX per cell at lower temperatures, but another experiment (Dias et al., 2002) showed that a different *Aphanizomenon* strain produced more STX at higher temperatures. Regarding nutrients, a recent *meta*-analysis suggested that STX per cell decreased under N limitation and increased under P limitation because STXs are N-rich molecules (Van de Waal et al., 2014), yet many culture studies showed STX production increased under low N concentrations (Aguilera et al., 2017; Cirés et al., 2017; Dias et al., 2002; Yunes et al., 2009). Many STX-producing genera are capable of N-fixation, which might explain why some studies showed no effect of N concentration on STX production (Vargas et al., 2019; Vico et al., 2016). The effect of light on STX production has been less studied. Carneiro et al. (2009) showed STX production was highest at 100 μmol photons/m^2^/s compared to 50 and 150 μmol photons/m^2^/s; however, Aguilera et al. (2017) showed no significant difference in STX production at 40 and 80 μmol photons/m^2^/s. These light levels represent a relatively narrow range and low intensity compared to full sunlight, which can be 2,000 μmol photons/m^2^/s on cloud-free days during mid-summer. Elevated carbon dioxide was shown to decrease STX quota but elevated carbon dioxide with elevated nitrate increased STX in laboratory experiments with *Dolichospermum* (Kramer et al., 2022). Interactions among these factors likely also influence STX production (Cirés et al., 2017; Kramer et al., 2022). Other studies have shown that ambient salt stress or alkalinity affected STX production (Carneiro et al., 2013; D’Agostino et al., 2016; Ongley et al., 2016). These mixed conclusions collectively suggest there might be species or strain-specific impacts or that the lack of compressive analytical methods and not accounting for interactions among factors in studies may be biasing general conclusions. Clearly, more research is needed on the drivers of STX production.

Benthic cyanobacteria biomass and STX responded conversely with the highest biomass in the shallow high-light level with extra P and ammonium, while STX was higher at the deeper lower-light level. Regarding biomass, enrichments of P and ammonium have shown to simulate non-N-fixing cyanobacteria in the plankton of Lake Erie (Chaffin et al., 2022, 2018), which agrees with the non-N-fixing taxa observed in our NDS experiments. Lake sediment phosphorus content, not water column P concentration, impacts where *Microseira* mats form (Putnam et al., 2022). Substrate type and physical forces (currents, waves) determine where benthic algae can colonize (Cantonati and Lowe, 2014; Vadeboncoeur et al., 2008, 2006). The opposing trends of biomass and STX suggest that the two processes are not correlated. Additionally, we observed significant interactions among depth (i.e., light), nutrients, and season (Table 2) in the NDS experiments, and interactions among factors has been document in SXT production in the plankton (Aguilera et al., 2017; Ciŕes et al., 2017; Kramer et al., 2022). As with the plankton, we cannot determine which benthic taxa were responsible for STX production.

Studying benthic algae is more challenging than phytoplankton due to its attachment as patches on substrates rather than being suspended more homogeneously in the water column (Wood et al., 2020). Stream ecologists frequently utilize NDS experiments (Tank et al., 2017), but relatively few studies have used NDS in lakes (Ozersky and Camilleri, 2021; Winfield Fairchild and Lowe, 1984). The drawback to the *in situ* NDS design is that only a few factors can be tested for compared to lab incubations. Additionally, the substrate type (frits, clay pots, filters) used in NDS affects which benthic algae colonize the experiments (Capps et al., 2011) and which analyses can be conducted (i.e., we could not extract DNA from the frits). NDS experiments can be useful for studying benthic cyanobacteria and toxin production, but their limitations must be accounted for in the experimental design.

## 5. Conclusion

In conclusion, STX and the STX biosynthesis gene *sxtA* were detected in varying amounts throughout the western basin of Lake Erie from 2018 to 2022. Analysis of three different datasets showed the patchy and ephemeral nature of *sxtA* in the water column, starkly contrasting with total cyanobacteria biomass and microcystin concentrations. Additionally, benthic cyanobacteria growth and *sxtA* in the water column were stimulated by phosphorus and ammonium, which is also the favored source of N for *Microcystis*. While scientists continue to debate about the need for dual nutrient management (Paerl et al., 2016b) vs. P-only management (Schindler et al., 2016), those debates only focus on water column algal blooms and do not consider benthic blooms. These debates would be more inclusive if benthic algae were considered. Overlooking benthic production may inhibit the complete understanding of saxitoxin and contributing sources, limiting management and monitoring strategies. With technological advancements and a better understanding of STX, it is essential for STX research and monitoring to not be overlooked when maintaining the safety of those utilizing Lake Erie. Additional research and continued monitoring are needed, and we emphasize that those future efforts should not solely focus on microcystins or the practice of seeing green and measuring only microcystins.

## Supplementary Material

appendix S1

## Figures and Tables

**Table 1:** The percentage of *sxtA* detections at 11 public water systems with source water in western Lake Erie between 2019 and 2022 during May through October collected at biweekly intervals, and their location on Fig. 1 map.

Public Water System	Fig. 1	2019	2020	2021	2022	TOTAL
Toledo	TOL	0.0 %	0.0 %	0.0 %	0.0 %	0.0 %
Oregon	ORE	0.0 %	0.0 %	0.0 %	0.0 %	0.0 %
Carroll	CAR	27.3 %	7.7 %	7.7 %	0.0 %	9.8 %
Ottawa County	OTT	23.1 %	0.0 %	7.1 %	0.0 %	7.4 %
Middle Bass Island	MBI	0.0 %	7.7 %	28.6 %	0.0 %	10.2 %
Put in Bay	PIB	18.2 %	0.0 %	16.7 %	7.1 %	10.0 %
Marblehead	MAR	41.7 %	0.0 %	0.0 %	7.1 %	11.8 %
Kelleys Island	KI	58.3 %	0.0 %	0.0 %	0.0 %	13.2 %
Camp Patmos	CP	20.0 %	Closed*	40.0 %	21.4 %	26.5 %
Sandusky	SAN	11.1 %	0.0 %	14.3 %	0.0 %	6.0 %
Huron	HUR	37.5 %	23.1 %	21.4 %	0.0 %	18.4 %
TOTAL		22.8 %	3.9 %	11.6 %	3.2 %	9.8 %

* Camp Patmos was closed in 2020 due to COVID-19.

**Table 2:** Summary statistics of the three-factor ANOVAs on the impact of season, depth, and nutrient treatment (abbreviated as s, z, and t, respectively) on benthic cyanobacteria and saxitoxin in six nutrient diffusing substrata experiments conducted during the summers of 2018 and 2019.

	Cyanobacteria-chl a						Saxitoxin				
		2018		2019				2018		2019	
Source	df	F	P	F	P	Source	df	F	P	F	P
Season (s)	2	251.0	<0.001	354.1	<0.001	Season (s)	2	109.1	<0.001	27.58	<0.001
Depth (z)	1	332.5	<0.001	139.4	<0.001	Depth (z)	1	55.11	<0.001	110.7	<0.001
Treatment (t)	5	44.23	<0.001	2.4	0.037	Treatment (t)	5	0.893	0.491	0.870	0.506
s * z	2	13.15	<0.001	16.6	<0.001	s * z	2	9.936	<0.001	15.95	<0.001
s * t	10	5.448	<0.001	3.3	<0.001	s * t	10	1.912	0.057	2.153	0.032
z * t	5	4.118	0.001	1.2	0.288	z * t	5	1.316	0.267	0.794	0.558
s * z * t	10	1.780	0.063	1.1	0.348	s * z * t	10	0.771	0.656	1.675	0.105

**Table 3:** Genera of benthic algae observed by microscopy in the nutrient-diffusing substrata experiments.

	Genera
July 2018	
Cyanobacteria	*Anabaena, Chroococcus, Merismopedia, Oscillatoria*
Green Algae, Desmids	*Ankistrodesmus, Cosmarium, Closterium, Pediastrum, Scenedesmus, Selenastrum, Stigeoclonium, Spirogrya*
Diatoms	*Cocconeis, Cymbella, Fragilaria, Gomphenema, Gomphoneis, Meloseria, Meridion, Navicula&Nitzschia*-like*, Rhoicosphenia
July 2019	
Cyanobacteria	*Leptolyngbya, Pseudanabaena*
Green Algae, Desmids	*Ankistrodesmus, Closterium, Pediastrum, Scenedesmus, Stigeoclonium*
Diatoms	*Cocconeis, Cymbella, Gomphenema, Meloseria, Navicula&Nitzschia*-like*, Rhoicosphenia
August 2018	
Cyanobacteria	*Chroococcus, Kamptonema, Leptolyngbya, Merismopedia, Microseira, Oscillatoria, Planktolyngbya, Pseudanabaena*
Green Algae, Desmids	*Ankistrodesmus, Closterium, Cosmarium, Oedogonium, Pandorina, Pediastrum, Scenedesmus, Spirogrya*
Diatoms	*Cocconeis, Gomphenema, Encyonema, Meloseria, Navicula&Nitzschia*-like*
August 2019	
Cyanobacteria	*Chroococcus, Kamptonema, Leptolyngbya, Merismopedia, Pseudanabaena*
Green Algae, Desmids	*Cosmarium, Oedogonium, Pediastrum, Scenedesmus, Spirogrya, Stigeoclonium*
Diatoms	*Cocconeis, Cymbella, Gomphenema, Gryosigma, Navicula&Nitzschia*-like*
September 2018	
Cyanobacteria	*Chroococcus, Kamptonema, Leptolyngbya, Merismopedia, Microcoleus, Microseira, Oscillatoria, Planktolyngbya, Phormidium, Pseudanabaena*
Green Algae, Desmids	*Coelastrum, Cosmarium, Mougeotia, Oocystis, Pediastrum, Scenedesmus, Stigeoclonium, Staurastrum*
Diatoms	*Cocconeis, Cymbella, Diatoma, Encytonema, Gryosigma, Meloseria, Navicula&Nitzschia*-like*

* *Navicula*-like and *Nitzschia*-like genera are difficult to differentiate and beyond the scope of our study.

## Data Availability

Data collected from sites MB18, WB83, and GIW are available at NOAA’s National Center for Environmental Information (NCEI Accession 0276941). Data obtained from the public water systems is available from Ohio EPA (https://epa.ohio.gov/divisions-and-offices/drinking-and-ground-waters/public-water-systems/harmful-algal-blooms). Experimental data can be made available upon request.

## References

[R1] Aguilera A, Aubriot L, Echenique RO, Salerno GL, Brena BM, Pírez M, Bonilla S, 2017. Synergistic effects of nutrients and light favor nostocales over non-heterocystous cyanobacteria. Hydrobiologia 794, 241–255. 10.1007/s10750-017-3099-1.

[R2] Al-Tebrineh J, Mihali TK, Pomati F, Neilan BA, 2010. Detection of saxitoxin-producing cyanobacteria and *Anabaena circinalis* in environmental water blooms by quantitative PCR. Appl. Environ. Microbiol 76, 7836–7842. 10.1128/AEM.00174-10. 20935128 PMC2988610

[R3] Al-Tebrineh J, Pearson LA, Yasar SA, Neilan BA, 2012. A multiplex qPCR targeting hepato- and neurotoxigenic cyanobacteria of global significance. Harmful Algae 15, 19–25. 10.1016/j.hal.2011.11.001.

[R4] Ballot A, Bernard C, Fastner J, 2016. Saxitoxin and analogues. In: Handbook of Cyanobacterial Monitoring and Cyanotoxin Analysis. John Wiley & Sons Ltd, pp. 148–154. 10.1002/9781119068761.ch14.

[R5] Barnard MA, Chaffin JD, Plaas HE, Boyer GL, Wei B, Wilhelm SW, Rossignol KL, Braddy JS, Bullerjahn GS, Bridgeman TB, Davis TW, Wei J, Bu M, Paerl HW, 2021. Roles of nutrient limitation on Western Lake Erie CyanoHAB toxin production. Toxins 13, 47. 10.3390/toxins13010047. 33435505 PMC7828104

[R6] Berry MA, Davis TW, Cory RM, Duhaime MB, Johengen TH, Kling GW, Marino JA, Den Uyl PA, Gossiaux D, Dick GJ, Denef VJ, 2017. Cyanobacterial harmful algal blooms are a biological disturbance to Western Lake Erie bacterial communities: bacterial community ecology of CHABs. Environ. Microbiol 19, 1149–1162. 10.1111/1462-2920.13640. 28026093

[R7] Bridgeman TB, Chaffin JD, Kane DD, Conroy JD, Panek SE, Armenio PM, 2012. From river to Lake: phosphorus partitioning and algal community compositional changes in Western Lake Erie. J. Gt. Lakes Res 38, 90–97. 10.1016/j.jglr.2011.09.010.

[R8] Bridgeman TB, Penamon WA, 2010. *Lyngbya wollei* in western Lake Erie. J. Gt. Lakes Res 36, 167–171. 10.1016/j.jglr.2009.12.003.

[R9] Burford MA, Carey CC, Hamilton DP, Huisman J, Paerl HW, Wood SA, Wulff A, 2020. Perspective: advancing the research agenda for improving understanding of cyanobacteria in a future of global change. Harmful Algae, Climate Change and Harmful Algal Blooms 91, 101601. 10.1016/j.hal.2019.04.004. 32057347

[R10] Cantonati M, Lowe RL, 2014. Lake benthic algae: toward an understanding of their ecology. Freshw. Sci 33, 475–486. 10.1086/676140.

[R11] Capps KA, Booth MT, Collins SM, Davison MA, Moslemi JM, El-Sabaawi RW, Simonis JL, Flecker AS, 2011. Nutrient diffusing substrata: a field comparison of commonly used methods to assess nutrient limitation. J. North Am. Benthol. Soc 30, 522–532. 10.1899/10-146.1.

[R12] Carneiro RL, dos Santos MEV, Pacheco ABF, Azevedo SMF, de O.e., 2009. Effects of light intensity and light quality on growth and circadian rhythm of saxitoxins production in Cylindrospermopsis raciborskii (cyanobacteria). J. Plankton Res 31, 481–488. 10.1093/plankt/fbp006.

[R13] Carneiro RL, Pacheco ABF, De Oliveira SMF, e Azevedo, 2013. Growth and saxitoxin production by Cylindrospermopsis raciborskii (cyanobacteria) Correlate with water Hardness. Mar. Drugs 11, 2949–2963. 10.3390/md11082949. 23955286 PMC3766875

[R14] Casero MC, Ballot A, Agha R, Quesada A, Ciŕes S, 2014. Characterization of saxitoxin production and release and phylogeny of sxt genes in paralytic shellfish poisoning toxin-producing *Aphanizomenon gracile*. Harmful Algae 37, 28–37. 10.1016/j.hal.2014.05.006.

[R15] Catherine Q, Susanna W, Isidora E-S, Mark H, Aurélie V, Jean-François H, 2013. A review of current knowledge on toxic benthic freshwater cyanobacteria – ecology, toxin production and risk management. Water Res. 47, 5464–5479. 10.1016/j.watres.2013.06.042. 23891539

[R16] Chaffin JD, Bridgeman TB, Bade DL, 2013. Nitrogen constrains the growth of late summer cyanobacterial blooms in Lake Erie. Adv. Microbiol 03, 16–26. 10.4236/aim.2013.36A003.

[R17] Chaffin JD, Davis TW, Smith DJ, Baer MM, Dick GJ, 2018. Interactions between nitrogen form, loading rate, and light intensity on *Microcystis* and *planktothrix* growth and microcystin production. Harmful Algae 73, 84–97. 10.1016/j.hal.2018.02.001. 29602509

[R18] Chaffin JD, Mishra S, Kane DD, Bade DL, Stanislawczyk K, Slodysko KN, Jones KW, Parker EM, Fox EL, 2019. Cyanobacterial blooms in the central basin of Lake Erie: potentials for cyanotoxins and environmental drivers. J. Gt. Lakes Res 45, 277–289. 10.1016/j.jglr.2018.12.006.

[R19] Chaffin JD, Bratton JF, Verhamme EM, Bair HB, Beecher AA, Binding CE, Birbeck JA, Bridgeman TB, Chang X, Crossman J, Currie WJS, Davis TW, Dick GJ, Drouillard KG, Errera RM, Frenken T, MacIsaac HJ, McClure A, McKay RM, Reitz LA, Domingo JWS, Stanislawczyk K, Stumpf RP, Swan ZD, Snyder BK, Westrick JA, Xue P, Yancey CE, Zastepa A, Zhou X, 2021. The Lake Erie HABs grab: a binational collaboration to characterize the western basin cyanobacterial harmful algal blooms at an unprecedented high-resolution spatial scale. Harmful Algae 108, 102080. 10.1016/j.hal.2021.102080. 34588116 PMC8682807

[R20] Chaffin JD, Westrick JA, Furr E, Birbeck JA, Reitz LA, Stanislawczyk K, Li W, Weber PK, Bridgeman TB, Davis TW, Mayali X, 2022. Quantification of microcystin production and biodegradation rates in the western basin of Lake Erie. Limnol. Oceanogr 67, 1470–1483. 10.1002/lno.12096. 36248197 PMC9543754

[R21] Christensen VG, Khan E, 2020. Freshwater neurotoxins and concerns for human, animal, and ecosystem health: a review of anatoxin-a and saxitoxin. Sci. Total Environ 736, 139515 10.1016/j.scitotenv.2020.139515. 32485372

[R22] Ciŕes S, Delgado A, González-Pleiter M, Quesada A, 2017. Temperature influences the production and transport of saxitoxin and the expression of *sxt* genes in the cyanobacterium *Aphanizomenon gracile*. Toxins 9, 322. 10.3390/toxins9100322. 29027918 PMC5666369

[R23] D’Agostino PM, Song X, Neilan BA, Moffitt MC, 2016. Proteogenomics of a saxitoxin-producing and non-toxic strain of Anabaena circinalis (cyanobacteria) in response to extracellular NaCl and phosphate depletion. Environ. Microbiol 18, 461–476. 10.1111/1462-2920.13131. 26568470

[R24] Dias E, Pereira P, Franca S, 2002. Production of Paralytic shellfish toxins by Aphanizomenon sp. lmecya 31 (cyanobacteria). J. Phycol 38, 705–712. 10.1046/j.1529-8817.2002.01146.x.

[R25] Gold Standard Diagnostics, 2022. ABRAXIS^®^ Saxitoxin (PSP) ELISA Microtiter Plate Enzyme-Linked Immunosorbent Assay for the Determination of Saxitoxin (PSP) in Water and Contaminated Samples. https://www.goldstandarddiagnostics.us/home/products/rapid-test-kits/algal-toxins/algal-toxin-elisa-plate-kits/abraxis-saxitoxins-psp-elisa-96-test/.

[R26] Golnick PC, Chaffin JD, Bridgeman TB, Zellner BC, Simons VE, 2016. A comparison of water sampling and analytical methods in western Lake Erie. J. Gt. Lakes Res 42, 965–971. 10.1016/j.jglr.2016.07.031.

[R27] Hašler P, Dvořák P, Johansen JR, Kitner M, Ondřej V, Poulíčková A, 2012. Morphological and molecular study of epipelic filamentous genera phormidium, microcoleus and geitlerinema (oscillatoriales, cyanophyta/cyanobacteria). Fottea 12, 341–356. 10.5507/fot.2012.024.

[R28] Hrycik AR, Shambaugh A, Stockwell JD, 2019. Comparison of FlowCAM and microscope biovolume measurements for a diverse freshwater phytoplankton community. J. Plankton Res fbz056 10.1093/plankt/fbz056.

[R29] Jankowiak J, Hattenrath-Lehmann T, Kramer BJ, Ladds M, Gobler CJ, 2019. Deciphering the effects of nitrogen, phosphorus, and temperature on cyanobacterial bloom intensification, diversity, and toxicity in western Lake Erie. Limnol. Oceanogr 64, 1347–1370. 10.1002/lno.11120.

[R30] Jørgensen NOG, Podduturi R, Michelsen CF, Jepsen T, Moraes M. de A.B., 2022. Fate of Saxitoxins in Lake Water: Preliminary Testing of Degradation by Microbes and Sunlight. Water 14, 3556. Doi: 10.3390/w14213556.

[R31] Kramer BJ, Davis TW, Meyer KA, Rosen BH, Goleski JA, Dick GJ, Oh G, Gobler CJ, 2018. Nitrogen limitation, toxin synthesis potential, and toxicity of cyanobacterial populations in Lake Okeechobee and the St. Lucie River Estuary, Florida, during the 2016 state of emergency event. PLoS ONE 13, e0196278. 10.1371/journal. pone.0196278. 29791446 PMC5965861

[R32] Kramer BJ, Hem R, Gobler CJ, 2022. Elevated CO2 significantly increases N2 fixation, growth rates, and alters microcystin, anatoxin, and saxitoxin cell quotas in strains of the bloom-forming cyanobacteria, *dolichospermum*. Harmful Algae 120, 102354. 10.1016/j.hal.2022.102354. 36470609

[R33] Laiveling A, Nauman C, Stanislawczyk K, Bair HB, Kane DD, Chaffin JD, 2022. Potamoplankton of the Maumee River during 2018 and 2019: the relationship between cyanobacterial toxins and environmental factors. J. Gt. Lakes Res 48, 1587–1598. 10.1016/j.jglr.2022.08.015. PMC11658212 39703700

[R34] Li X, Dreher TW, Li R, 2016. An overview of diversity, occurrence, genetics and toxin production of bloom-forming dolichospermum (anabaena) species. Harmful algae, global expansion of Harmful cyanobacterial blooms. Diversity, Ecology, Causes, and Controls 54, 54–68. 10.1016/j.hal.2015.10.015. 28073482

[R35] Llewellyn LE, Negri AP, Doyle J, Baker PD, Beltran EC, Neilan BA, 2001. Radioreceptor assays for sensitive detection and quantitation of saxitoxin and its analogues from strains of the freshwater cyanobacterium, *Anabaena circinalis*. Environ. Sci. Technol 35, 1445–1451. 10.1021/es001575z. 11348083

[R36] Loftin KA, Graham JL, Hilborn ED, Lehmann SC, Meyer MT, Dietze JE, Griffith CB, 2016. Cyanotoxins in inland lakes of the United States: occurrence and potential recreational health risks in the EPA National Lakes Assessment 2007. Harmful Algae 56, 77–90. 10.1016/j.hal.2016.04.001. 28073498

[R37] McAllister TG, Wood SA, Hawes I, 2016. The rise of toxic benthic phormidium proliferations: a review of their taxonomy, distribution, toxin content and factors regulating prevalence and increased severity. Harmful Algae 55, 282–294. 10.1016/j.hal.2016.04.002. 28073542

[R38] McGregor GB, Sendall BC, 2015. Phylogeny and toxicology of Lyngbya wollei (cyanobacteria, oscillatoriales) from north-eastern Australia, with a description of microseira gen. nov. J. Phycol 51, 109–119. 10.1111/jpy.12256. 26986262

[R39] Meriluoto J, Spoof L, Codd GA, 2016. Handbook of cyanobacterial monitoring and cyanotoxin analysis. John Wiley & Sons Ltd.

[R40] Michalak AM, Anderson EJ, Beletsky D, Boland S, Bosch NS, Bridgeman TB, Chaffin JD, Cho K, Confesor R, Daloğlu I, DePinto JV, Evans MA, Fahnenstiel GL, He L, Ho JC, Jenkins L, Johengen TH, Kuo KC, LaPorte E, Liu X, McWilliams MR, Moore MR, Posselt DJ, Richards RP, Scavia D, Steiner AL, Verhamme E, Wright DM, Zagorski MA, 2013. Record-setting algal bloom in Lake Erie caused by agricultural and meteorological trends consistent with expected future conditions. Proc. Natl. Acad. Sci 110, 6448–6452. 10.1073/pnas.1216006110. 23576718 PMC3631662

[R41] O’Neil JM, Davis TW, Burford MA, Gobler CJ, 2012. The rise of harmful cyanobacteria blooms: the potential roles of eutrophication and climate change. Harmful Algae, Harmful Algae-the Requirement for Species-Specific Information 14, 313–334. 10.1016/j.hal.2011.10.027.

[R42] Ongley SE, Pengelly JJL, Neilan BA, 2016. Elevated Na+ and pH influence the production and transport of saxitoxin in the cyanobacteria Anabaena circinalis AWQC131C and Cylindrospermopsis raciborskii T3. Environ. Microbiol 18, 427–438. 10.1111/1462-2920.13048. 26347118

[R43] Ozersky T, Camilleri A, 2021. Factors regulating lake periphyton biomass and nutrient limitation status across a large trophic gradient. Freshw. Biol 66, 2338–2350. 10.1111/fwb.13836.

[R44] Paerl HW, Gardner WS, Havens KE, Joyner AR, McCarthy MJ, Newell SE, Qin B, Scott JT, 2016a. Mitigating cyanobacterial harmful algal blooms in aquatic ecosystems impacted by climate change and anthropogenic nutrients. Harmful Algae, Global Expansion of Harmful Cyanobacterial Blooms: Diversity, Ecology, Causes, and Controls 54, 213–222. 10.1016/j.hal.2015.09.009. 28073478

[R45] Paerl HW, Scott JT, McCarthy MJ, Newell SE, Gardner WS, Havens KE, Hoffman DK, Wilhelm SW, Wurtsbaugh WA, 2016b. It takes two to tango: when and where dual nutrient (n & p) reductions are needed to protect lakes and downstream ecosystems. Environ. Sci. Technol 50, 10805–10813. 10.1021/acs.est.6b02575. 27667268

[R46] Pearson LA, Dittmann E, Mazmouz R, Ongley SE, D’Agostino PM, Neilan BA, 2016. The genetics, biosynthesis and regulation of toxic specialized metabolites of cyanobacteria. Harmful Algae, Global Expansion of Harmful Cyanobacterial Blooms: Diversity, Ecology, Causes, and Controls 54, 98–111. 10.1016/j.hal.2015.11.002. 28073484

[R47] Podduturi R, Schlüter L, Liu T, Osti JAS, de M, Moraes AB, Jørgensen NOG, 2021. Monitoring of saxitoxin production in lakes in Denmark by molecular, chromatographic and microscopic approaches. Harmful Algae 101966. 10.1016/j.hal.2020.101966. 33526182

[R48] Pokrzywinski KL, Volk K, Rycroft TE, Wood S, Davis T, Lazorchak J, 2021. Aligning research and monitoring priorities for benthic cyanobacteria and cyanotoxins : a workshop summary (Report). Environmental Laboratory (U.S.).

[R49] Putnam SP, Smith ML, Metz TT, Womer AM, Sellers EJ, McClain SJ, Crandell CA, Scott GI, Shaw TJ, Ferry JL, 2022. Growth of the harmful benthic cyanobacterium *microseira wollei* is driven by legacy sedimentary phosphorous. Harmful Algae 117, 102263. 10.1016/j.hal.2022.102263. 35944964

[R50] Raabová L, Kovacik L, Elster J, Strunecký O, 2019. Review of the genus phormidesmis (cyanobacteria) based on environmental, morphological, and molecular data with description of a new genus leptodesmis. Phytotaxa 395, 1–16. 10.11646/phytotaxa.395.1.1.

[R51] Rinta-Kanto JM, Konopko EA, DeBruyn JM, Bourbonniere RA, Boyer GL, Wilhelm SW, 2009. Lake Erie *Microcystis*: relationship between microcystin production, dynamics of genotypes and environmental parameters in a large lake. Harmful Algae 8, 665–673. 10.1016/j.hal.2008.12.004.

[R52] Savela H, Spoof L, Perälä N, Preede M, Lamminmäki U, Nybom S, Häggqvist K, Meriluoto J, Vehniäinen M, 2015. Detection of cyanobacterial sxt genes and paralytic shellfish toxins in freshwater lakes and brackish waters on Åland Islands, Finland. Harmful Algae 46, 1–10. 10.1016/j.hal.2015.04.005.

[R53] Schindler DW, Carpenter SR, Chapra SC, Hecky RE, Orihel DM, 2016. Reducing phosphorus to curb lake eutrophication is a success. Environ. Sci. Technol 50, 8923–8929. 10.1021/acs.est.6b02204. 27494041

[R54] Shishido TK, Delbaje E, Wahlsten M, Vuori I, Jokela J, Gugger M, Fiore MF, Fewer DP, 2023. A cylindrospermopsin-producing cyanobacterium isolated from a microbial mat in the Baltic Sea. Toxicon 232, 107205. 10.1016/j.toxicon.2023.107205. 37406865

[R55] Smith ZJ, Martin RM, Wei B, Wilhelm SW, Boyer GL, 2019. Spatial and temporal Variation in Paralytic shellfish toxin production by benthic microseira (lyngbya) wollei in a freshwater New York Lake. Toxins 11, 44. 10.3390/toxins11010044. 30650549 PMC6356249

[R56] Steffen MM, Davis TW, McKay RML, Bullerjahn GS, Krausfeldt LE, Stough JMA, Neitzey ML, Gilbert NE, Boyer GL, Johengen TH, Gossiaux DC, Burtner AM, Palladino D, Rowe MD, Dick GJ, Meyer KA, Levy S, Boone BE, Stumpf RP, Wynne TT, Zimba PV, Gutierrez D, Wilhelm SW, 2017. Ecophysiological examination of the Lake Erie *Microcystis* bloom in 2014: linkages between biology and the water supply shutdown of Toledo. OH. Environ. Sci. Technol 51, 6745–6755. 10.1021/acs.est.7b00856. 28535339

[R57] Strunecký O, Komárek J, Johansen J, Lukěsová A, Elster J, 2013. Molecular and morphological criteria for revision of the genus microcoleus (oscillatoriales, cyanobacteria). J. Phycol 49, 1167–1180. 10.1111/jpy.12128. 27007635

[R58] Strunecký O, Komárek J, Smarda J, 2014. Kamptonema (microcoleaceae, cyanobacteria), a new genus derived from the polyphyletic phormidium on the basis of combined molecular and cytomorphological markers. Preslia 86, 193–207.

[R59] Strunecký O, Ivanova AP, Mareš J, 2023. An updated classification of cyanobacterial orders and families based on phylogenomic and polyphasic analysis. J. Phycol 59, 12–51. 10.1111/jpy.13304. 36443823

[R60] Tank J, Reisinger A, J. Rosi E, 2017. Nutrient Limitation and Uptake. In: Methods in Stream Ecology (pp.147–171) Doi: 10.1016/B978-0-12-813047-6.00009-7.

[R61] Tavakoli Y, Mohammadipanah F, Te SH, You L, Gin K-Y-H, 2021. Biodiversity, phylogeny and toxin production profile of cyanobacterial strains isolated from lake latyan in Iran. Harmful Algae 106, 102054. 10.1016/j.hal.2021.102054. 34154781

[R62] Vadeboncoeur Y, Kalff J, Christoffersen K, Jeppesen E, 2006. Substratum as a driver of variation in periphyton chlorophyll and productivity in lakes. J. North Am. Benthol. Soc 25, 379–392. 10.1899/0887-3593(2006)25[;379:SAADOV];2.0.CO;2.

[R63] Vadeboncoeur Y, Peterson G, Vander Zanden MJ, Kalff J, 2008. Benthic algal production across Lake size gradients: Interactions among morphometry, nutrients, and light. Ecology 89, 2542–2552. 10.1890/07-1058.1. 18831175

[R64] Van de Waal DB, Smith VH, Declerck SAJ, Stam ECM, Elser JJ, 2014. Stoichiometric regulation of phytoplankton toxins. Ecol. Lett 17, 736–742. 10.1111/ele.12280. 24712512

[R65] Vargas SR, dos Santos PV, Bottino F, Calijuri M, do C, 2019. Effect of nutrient concentration on growth and saxitoxin production of Raphidiopsis raciborskii (cyanophyta) interacting with Monoraphidium contortum (chlorophyceae). J. Appl. Phycol 10.1007/s10811-019-01972-w.

[R66] Vico P, Aubriot L, Martigani F, Rigamonti N, Bonilla S, Piccini C, 2016. Influence of nitrogen availability on the expression of genes involved in the biosynthesis of saxitoxin and analogs in *Cylindrospermopsis raciborskii*. Harmful Algae 56, 37–43. 10.1016/j.hal.2016.04.008. 28073495

[R67] Vinogradova OM, Nuriyeva MA, 2020. Missed taxon: on the generic affiliation of cyanobacteria *Oscillatoria tanganyikae* var. *caspica* usachev. Int. J. Algae 22. 10.1615/InterJAlgae.v22.i3.40.

[R68] Wehr JD, Sheath RG, Kociolek JP, 2014. Freshwater Algae of North America - 2nd Ed.

[R69] Wiese M, D’Agostino PM, Mihali TK, Moffitt MC, Neilan BA, 2010. Neurotoxic alkaloids: saxitoxin and its analogs. Mar. Drugs 8, 2185–2211. 10.3390/md8072185. 20714432 PMC2920551

[R70] Winfield Fairchild G, Lowe RL, 1984. Artificial substrates which release nutrients: effects on periphyton and invertebrate succession. Hydrobiologia 114, 29–37. 10.1007/BF00016599.

[R71] Wood S, Kelly L, Bouma-Gregson K, Humbert J-F, Laughinghouse IV H, Lazorchak J, McAllister T, McQueen A, Pokrzywinski K, Puddick J, Quiblier C, Reitz L, Ryan K, Vadeboncoeur Y, Zastepa A, Davis T, 2020. Toxic benthic freshwater cyanobacterial proliferations: challenges and solutions for enhancing knowledge and improving monitoring and mitigation. Freshw. Biol 65, 1–19. 10.1111/fwb.13532. PMC8715960 34970014

[R72] Yancey CE, Kiledal EA, Chaganti SR, Denef VJ, Errera RM, Evans JT, Hart LN, Isailovic D, James WS, Kharbush JJ, Kimbrel JA, Li W, Mayali X, Nitschky H, Polik CA, Powers MA, Premathilaka SH, Rappuhn NA, Reitz LA, Rivera SR, Zwiers CC, Dick GJ, 2023a. The Western Lake Erie culture collection: a promising resource for evaluating the physiological and genetic diversity of *Microcystis* and its associated microbiome. Harmful Algae 126, 102440. 10.1016/j.hal.2023.102440. 37290887

[R73] Yancey CE, Mathiesen O, Dick GJ, 2023b. Transcriptionally active nitrogen fixation and biosynthesis of diverse secondary metabolites by *dolichospermum* and *Aphanizomenon*-like cyanobacteria in western Lake Erie Microcystis blooms. Harmful Algae 124, 102408. 10.1016/j.hal.2023.102408. 37164563

[R74] Yunes JS, Rocha SDL, Giroldo D, Silveira S.B. da, Comin R, Bicho M. da S., Melcher SS, Sant’anna CL, Vieira AAH, 2009. Release of Carbohydrates and Proteins by a Subtropical Strain of *Raphidiopsis brookii* (cyanobacteria) Able to Produce Saxitoxin at Three Nitrate Concentrations1. J. Phycol 45, 585–591. Doi: 10.1111/j.1529-8817.2009.00673.x. 27034034

