# Supplementary material for: The spatiotemporal distribution of potential saxitoxin-producing cyanobacteria in western Lake Erie
Source: J Great Lakes Res. Author manuscript; Available in PMC 2024 Dec 19. (PMC11658238; doi:10.1016/j.jglr.2024.102342)

Electronic Supplementary Material Appendix S1
Cyanobacteria images from the nutrient diffusing substrata experiments.

The spatiotemporal distribution of the potential saxitoxin-producing cyanobacteria in western Lake Erie.

Callie Nauman[1,a], Keara Stanislawczyk[2], Laura A. Rietz[1,b], Justin D. Chaffin[2]*

1: Biological Sciences, Bowling Green State University, Bowling Green, OH, USA
2: F.T Stone Laboratory, The Ohio State University, 878 Bayview Ave. Put-in-Bay, OH 43456, USA

a Present address: Ohio EPA Division of Drinking and Ground Water. Columbus, OH, USA
b Present address: Department of Earth and Environmental Sciences, University of Michigan, 2534 North University Building, 1100 North University Avenue, Ann Arbor, MI 48109-1005, USA

* Corresponding Author: Chaffin.46@osu.edu

Image notes:

All cyanobacteria were identified with the keys in Komarek and Johansen (2015). Chapter 4 - Filamentous Cyanobacteria. In Freshwater Algae of North America (Second Edition). Edited by Wehr, Sheath, and Kocoilek.

Taxonomy changes since the publication of this book are noted in the text. Molecular analysis may give different identifications.

All images were captured with Olympus EP50 Camera on an Olympus CX23 Microscope and with Olympus EP View software.

Anabaena and several eukaryotic algae. 400x. From the June 2018 NDS. *Anabaena* white arrow. Two *Scenedesmus* species black arrow. *Cocconeis* red arrow. Fragilaria is also visible in the center of the image.

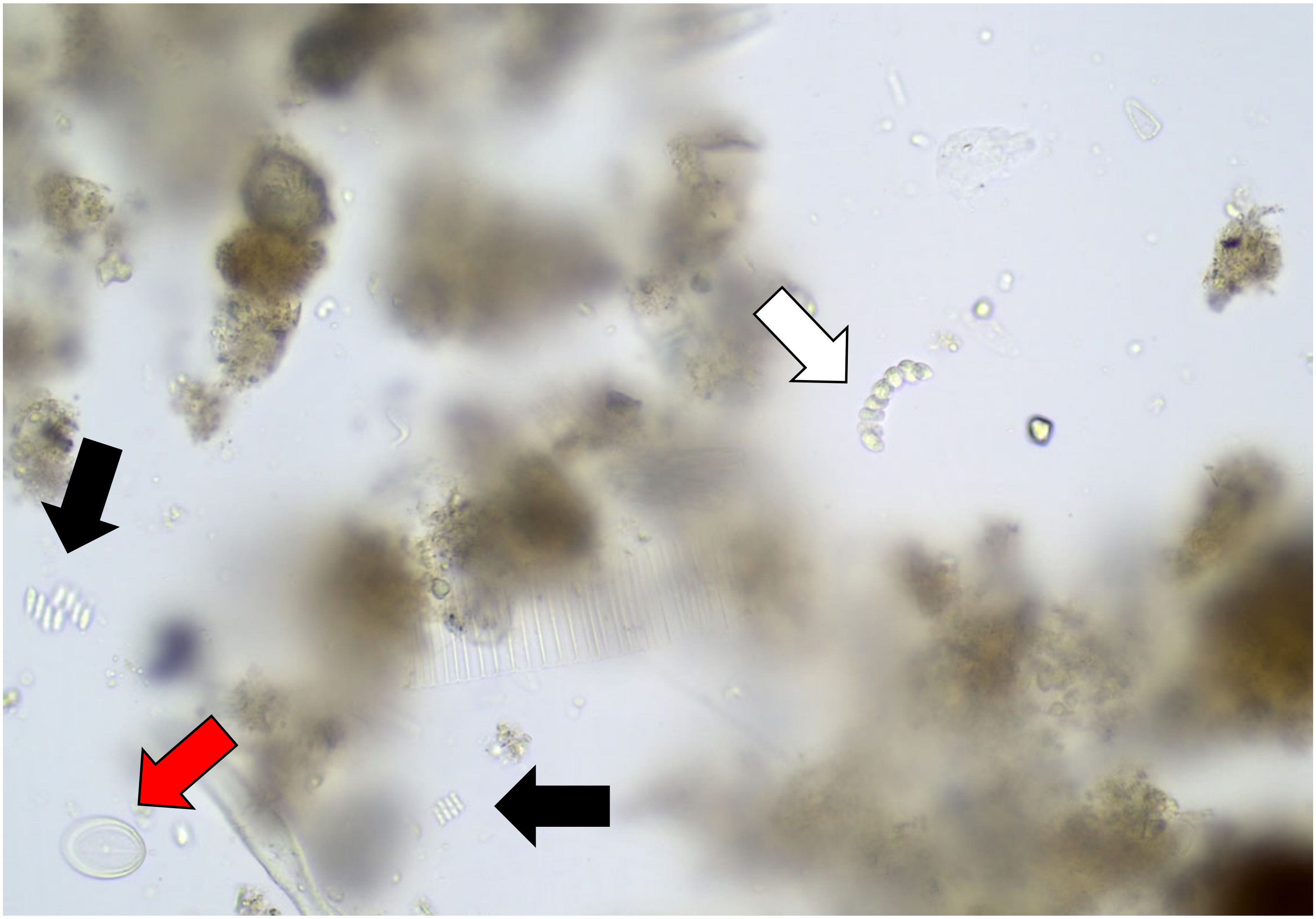

Eukaryotic algae from the June 2018 NDS. *Gomphenema* and green algae. 400x. *Gomphenema* white arrow. Two *Scenedesmus* species black arrow. Multiple examples of *Selenastrum* (red arrow) are also in the image.

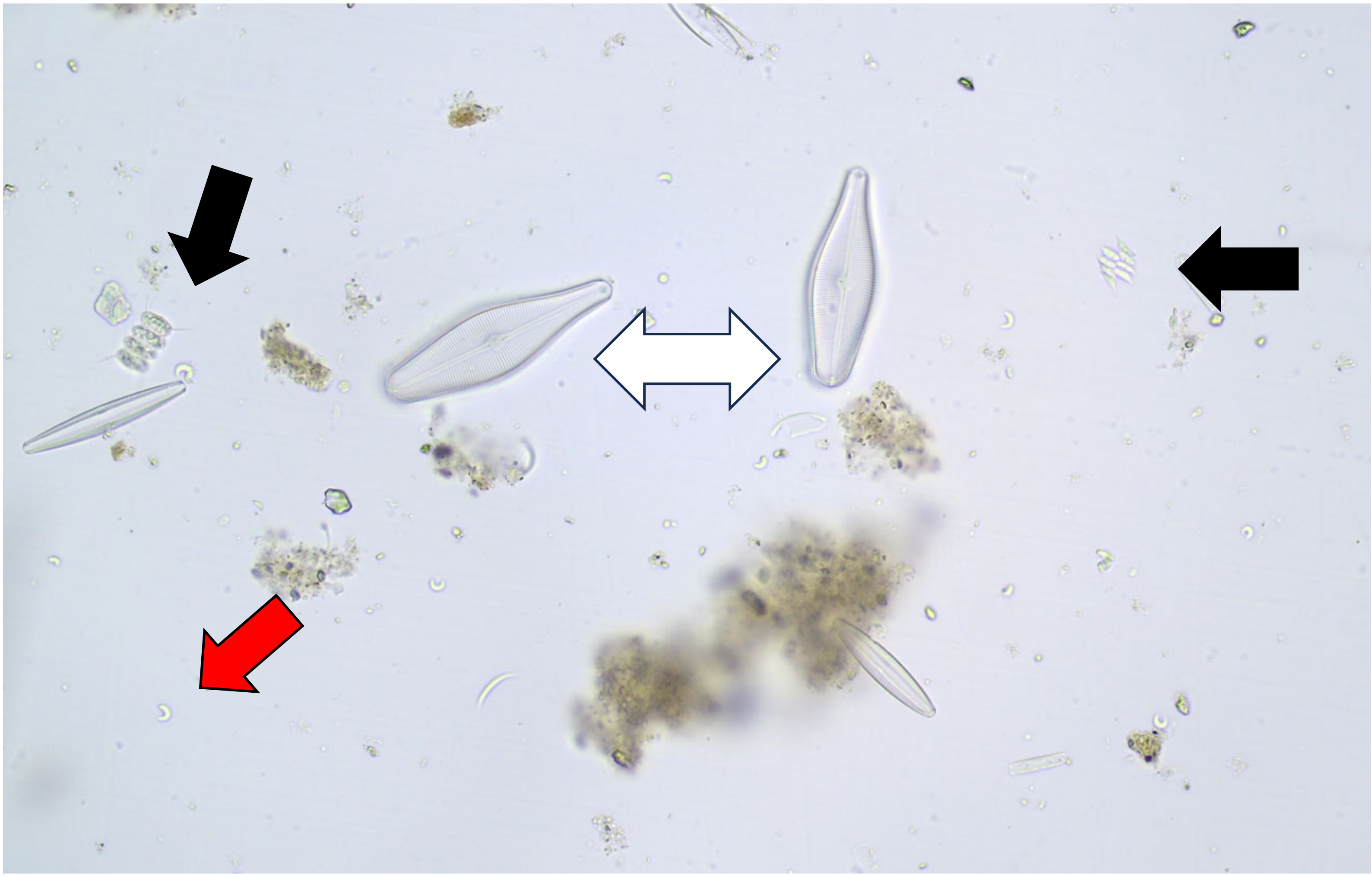

Pseudanabaena 400x. Diatoms and green algae are also in the images. From the August 2018 NDS. Note that the filaments lack a sheath and cells are longer than wide to isodiametric.

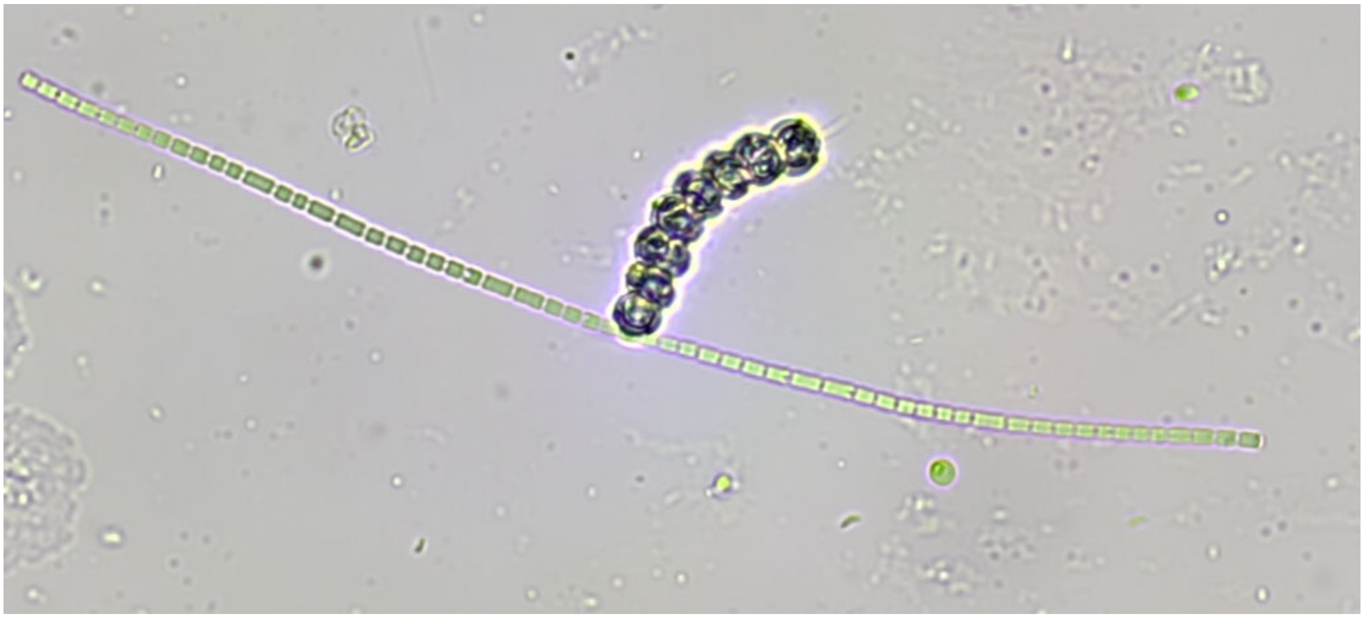

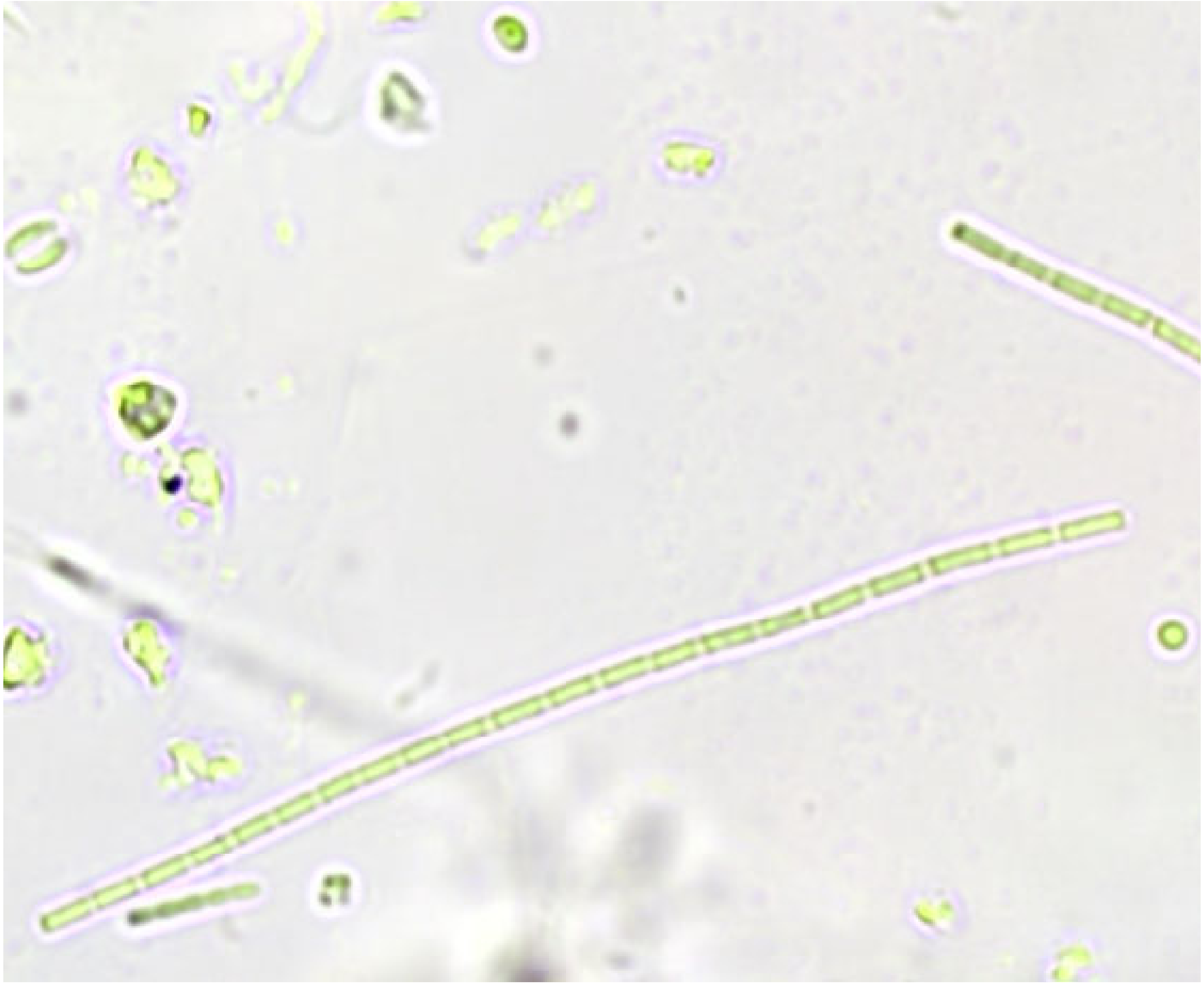

Leptolyngbya mat at 100x and 400x (zoomed in on the end of a filament). Several green algae are also in the image. From the August 2018 NDS. Note the thinness of the filaments, the sheath, and cells wider than long.

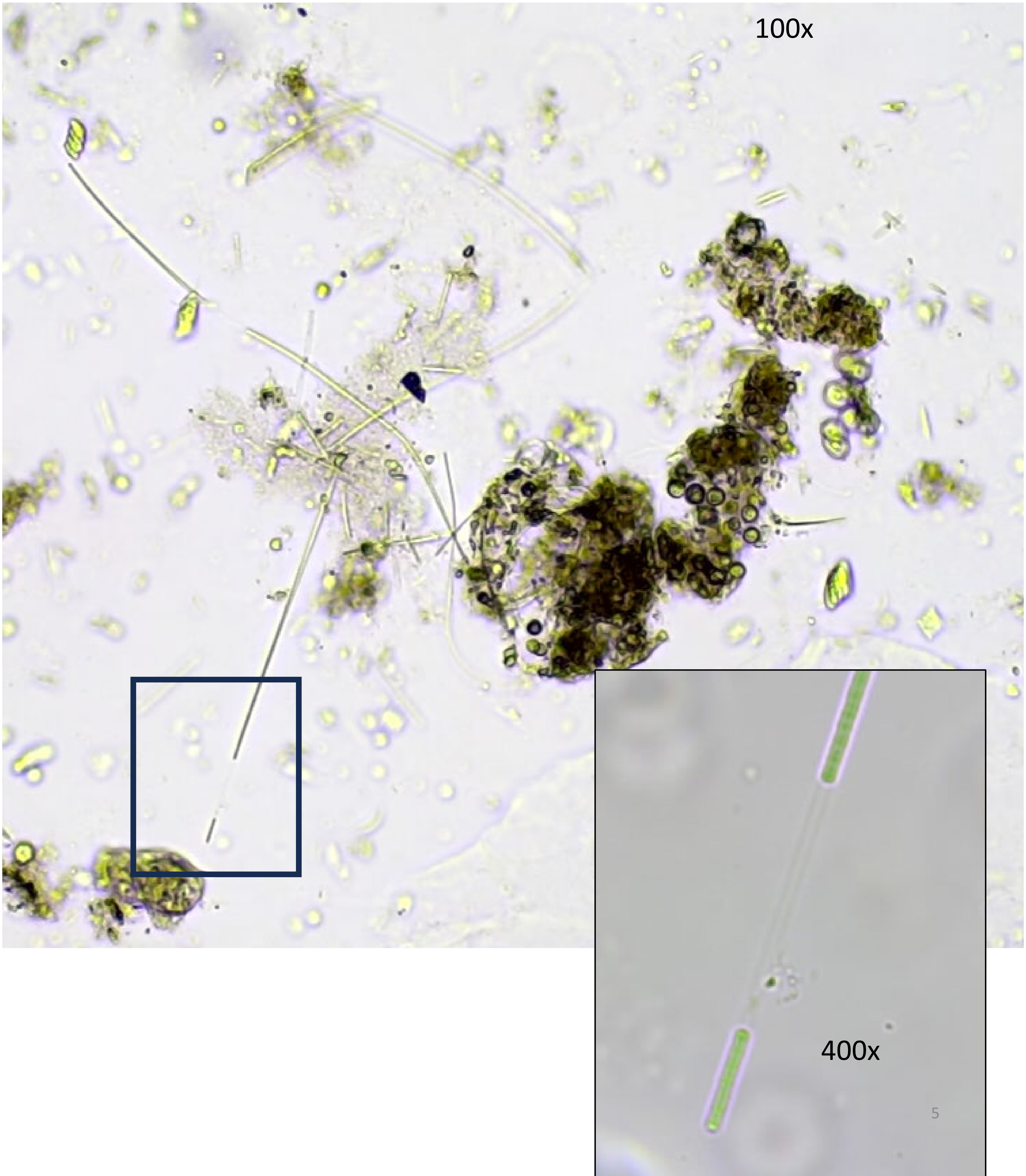

Leptolyngbya 400x. Two images at different focal points were pieced together for this display.
From the August 2018 NDS

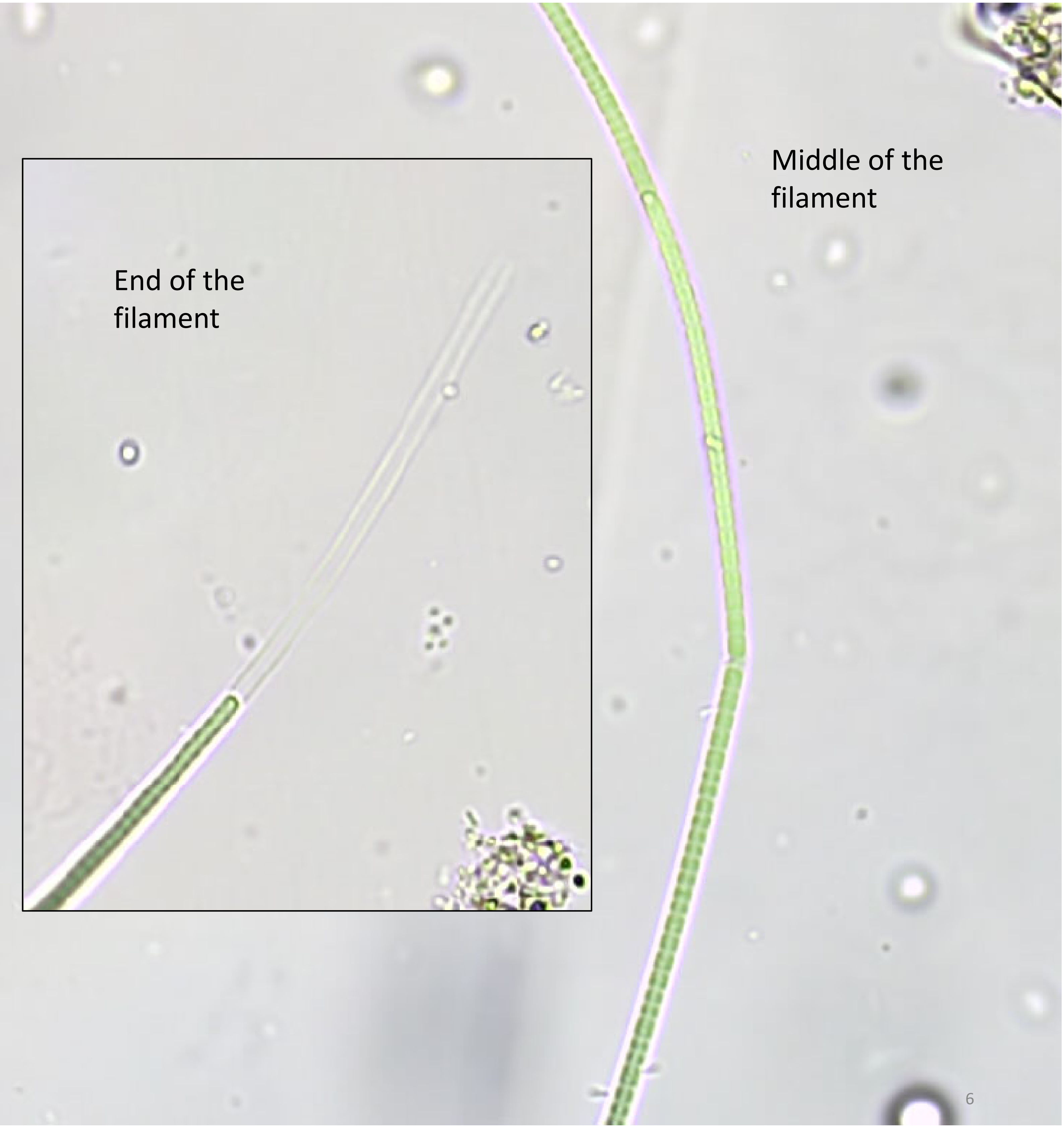

Leptolyngbya and *Scenedesmus.* 400x. From the September 2018 NDS. Note that the end cell morphology differs from the previous two specimens (pages 5 and 6), which is a characteristic of *Leptolyngyba*.

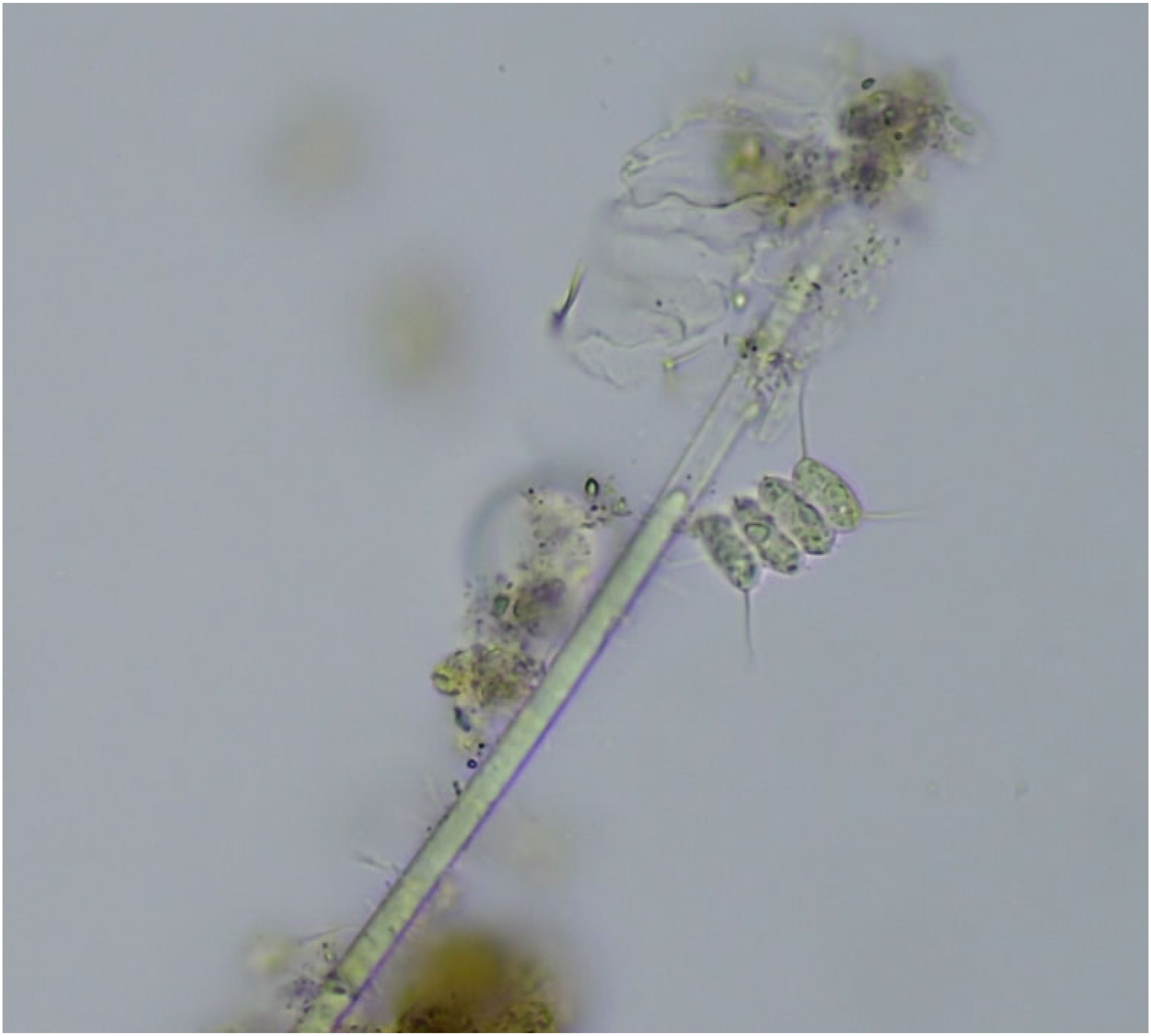

Leptolyngbya (black arrow), *Pseudanabaena* (white arrow), *Meloseira* and other diatoms.
400x. From the September 2018 NDS

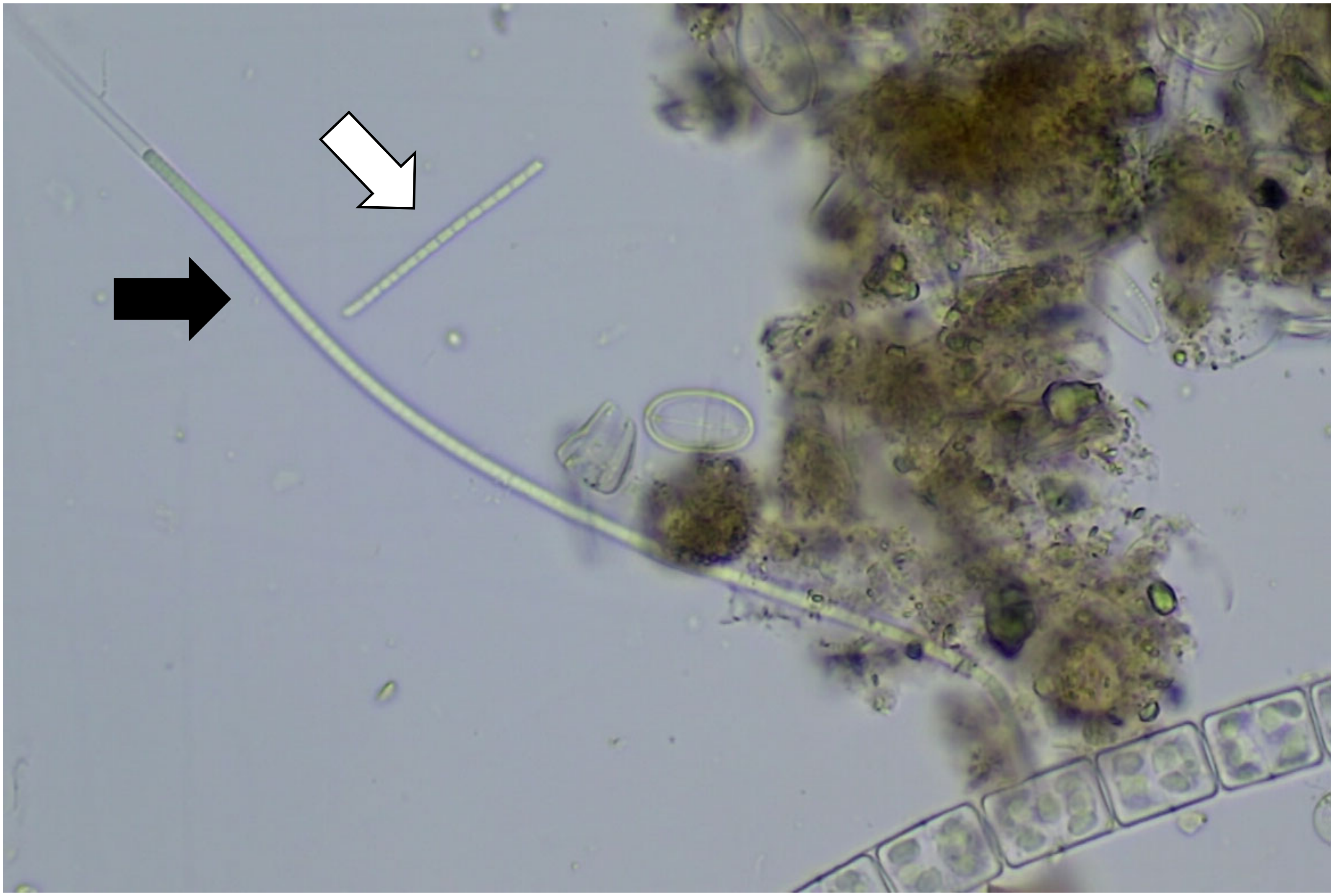

Oscillatoria 400x. Two images at different focal points were pieced together for this display. From the September 2018 NDS. Note the lack of a sheath and cell width is much longer than length, and the filament is much wider than *Pseudanabaena* and *Leptolyngbya*.

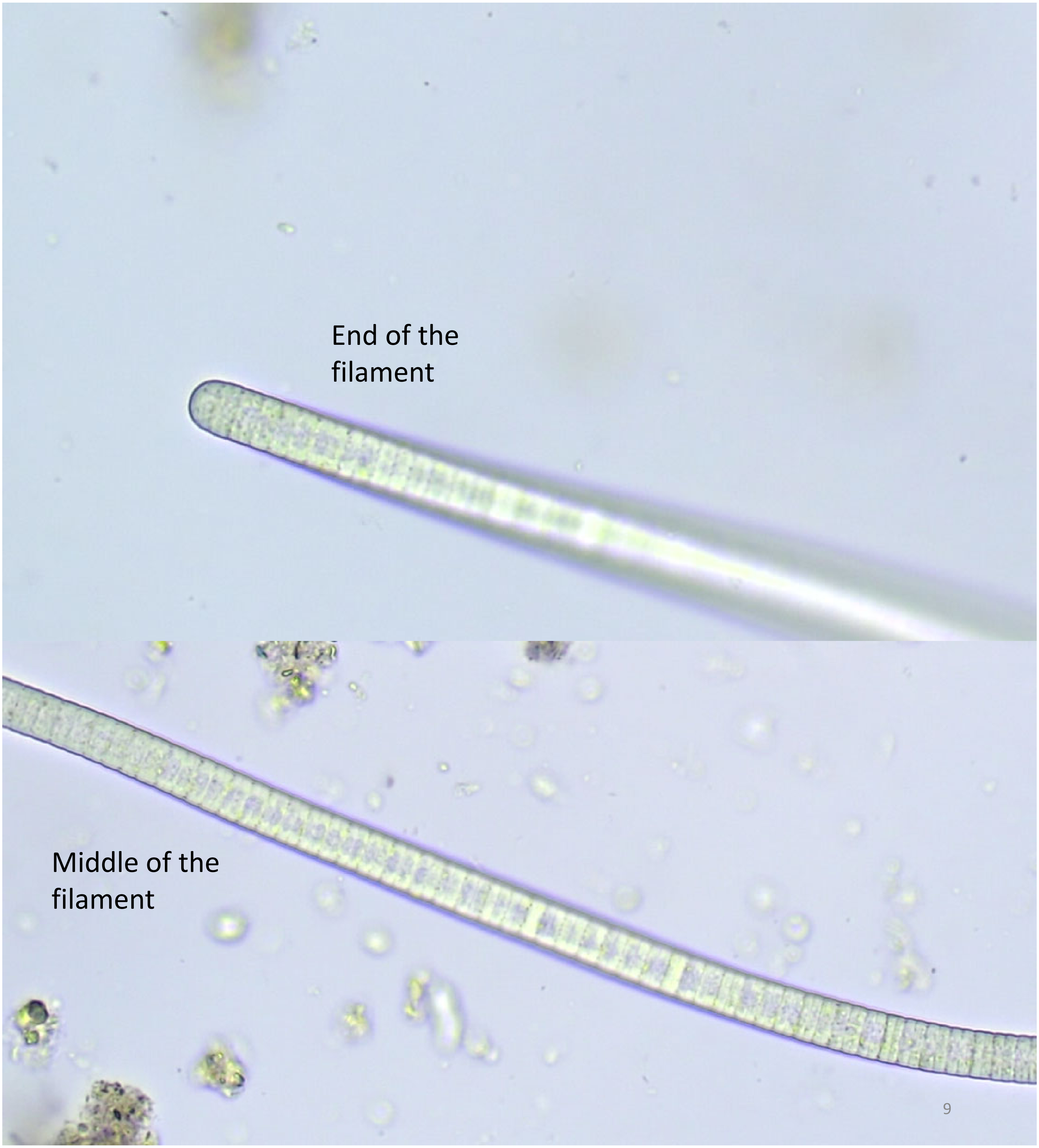

Phormidium mat at 40x and a zoomed view of the end of a filament at 400x. Note the sheath in the 400x view. Debris and epiphytes covered the filaments closer to the center of the mat. From the September 2018 NDS. Note the thin sheath and the cells are roughly isodiametric in the 400x image.

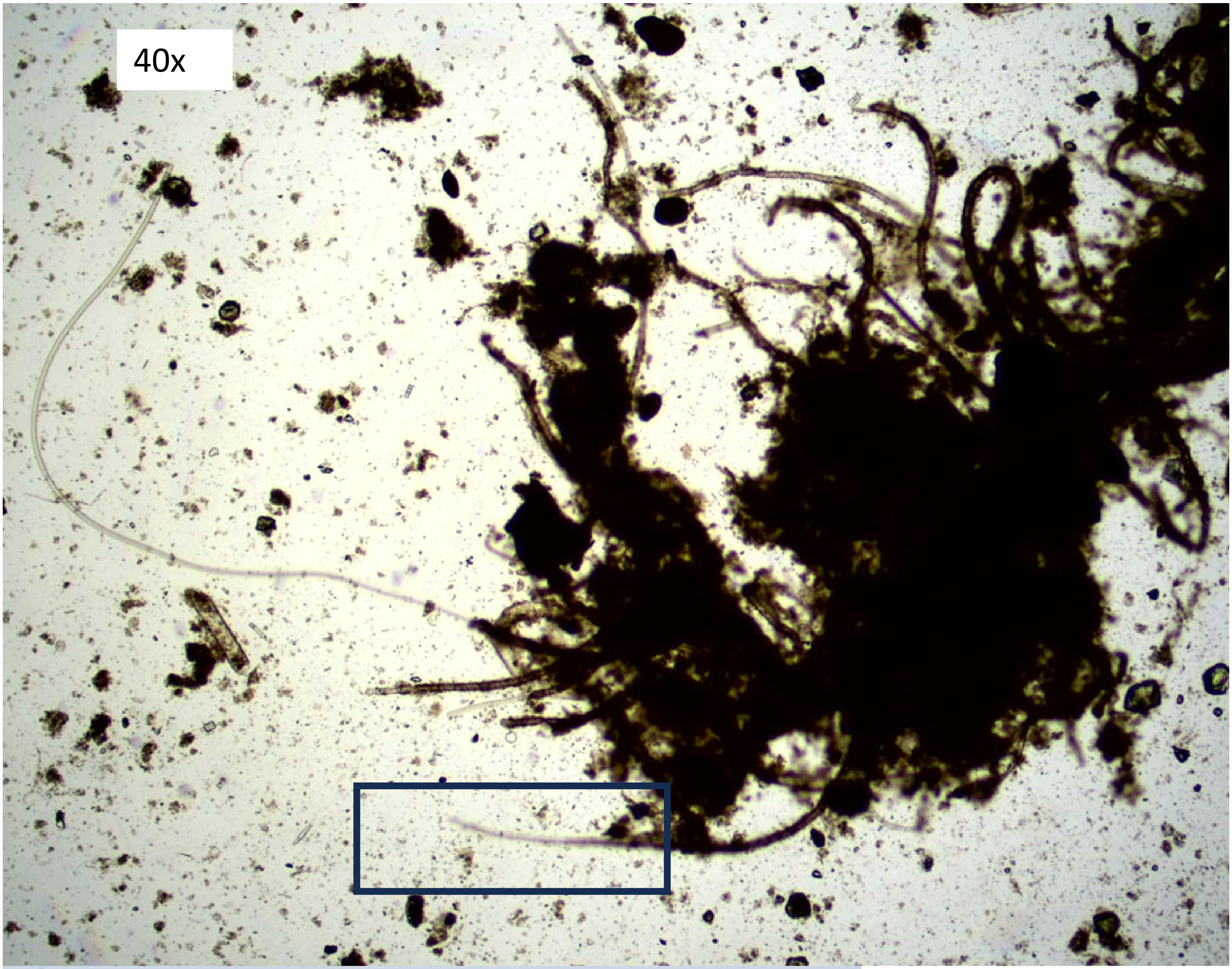

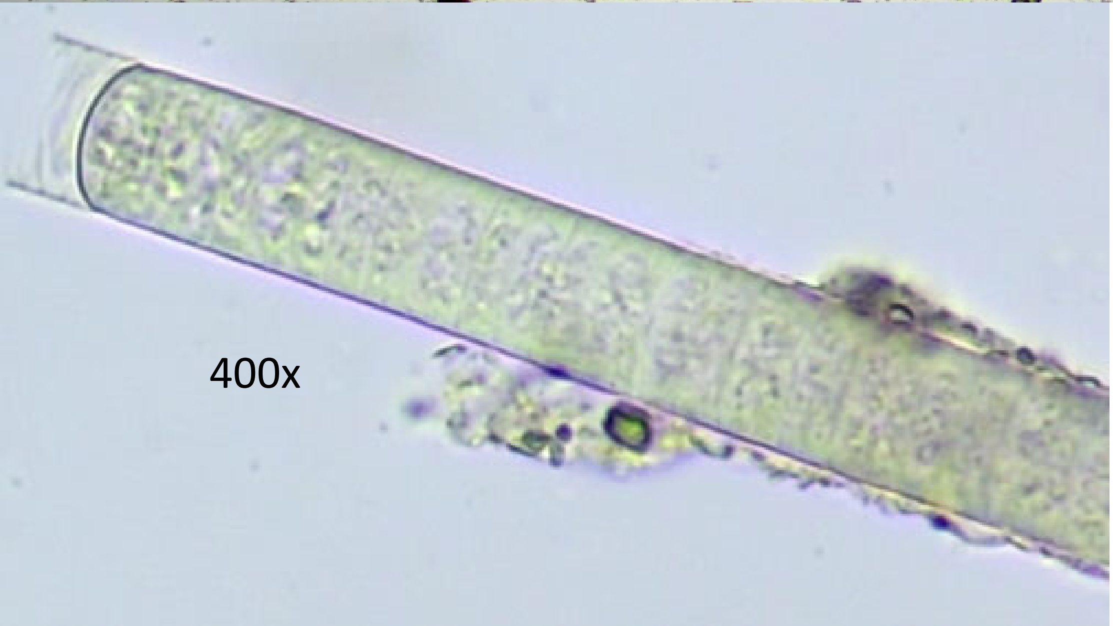

Microcoleus 400x. The end of the filament. From the September 2018 NDS

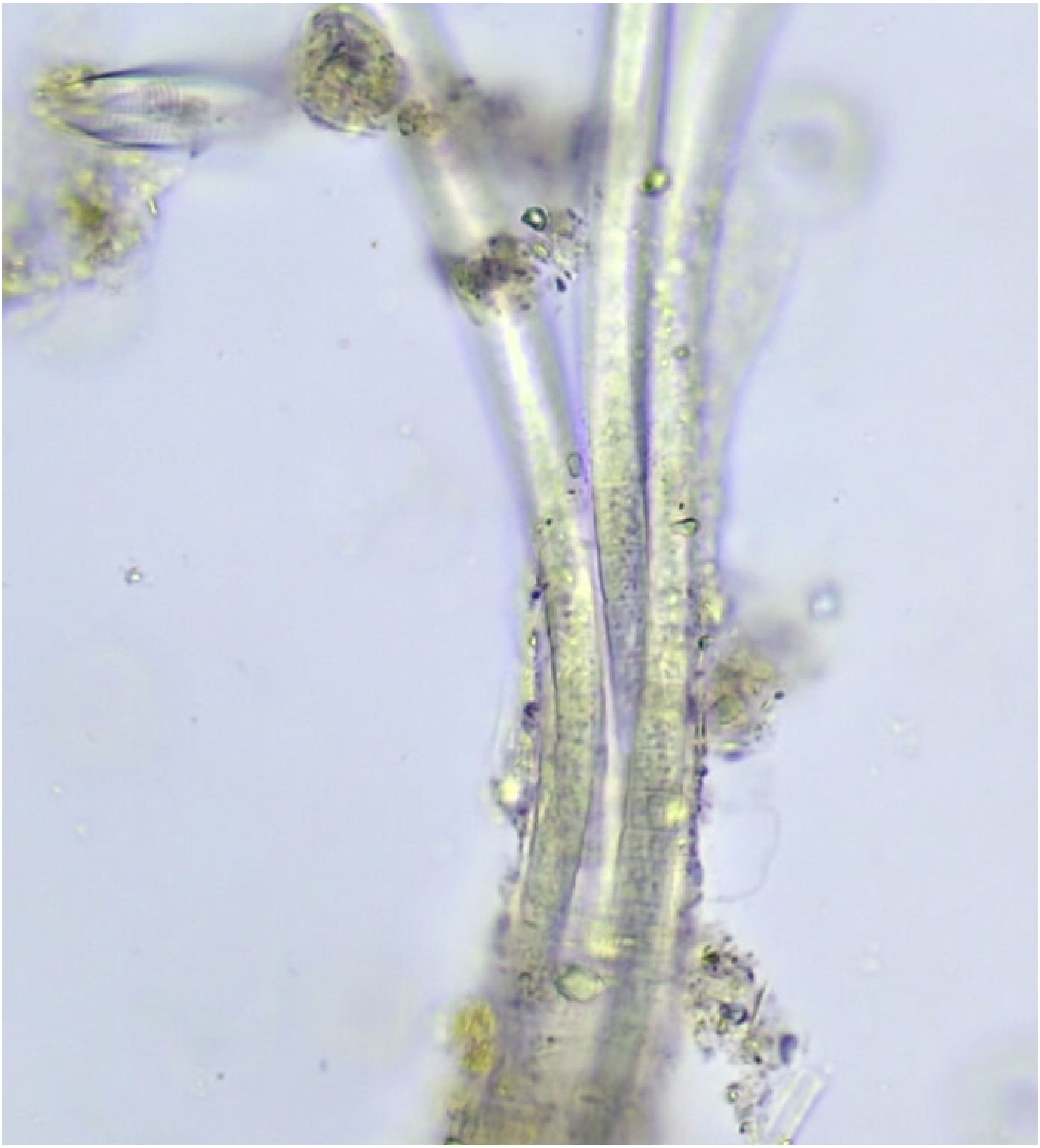

Microcoleus 400x. The same filament as the previous image but the middle of the filament. From the September 2018 NDS. Note the multiple trichomes within one sheath.

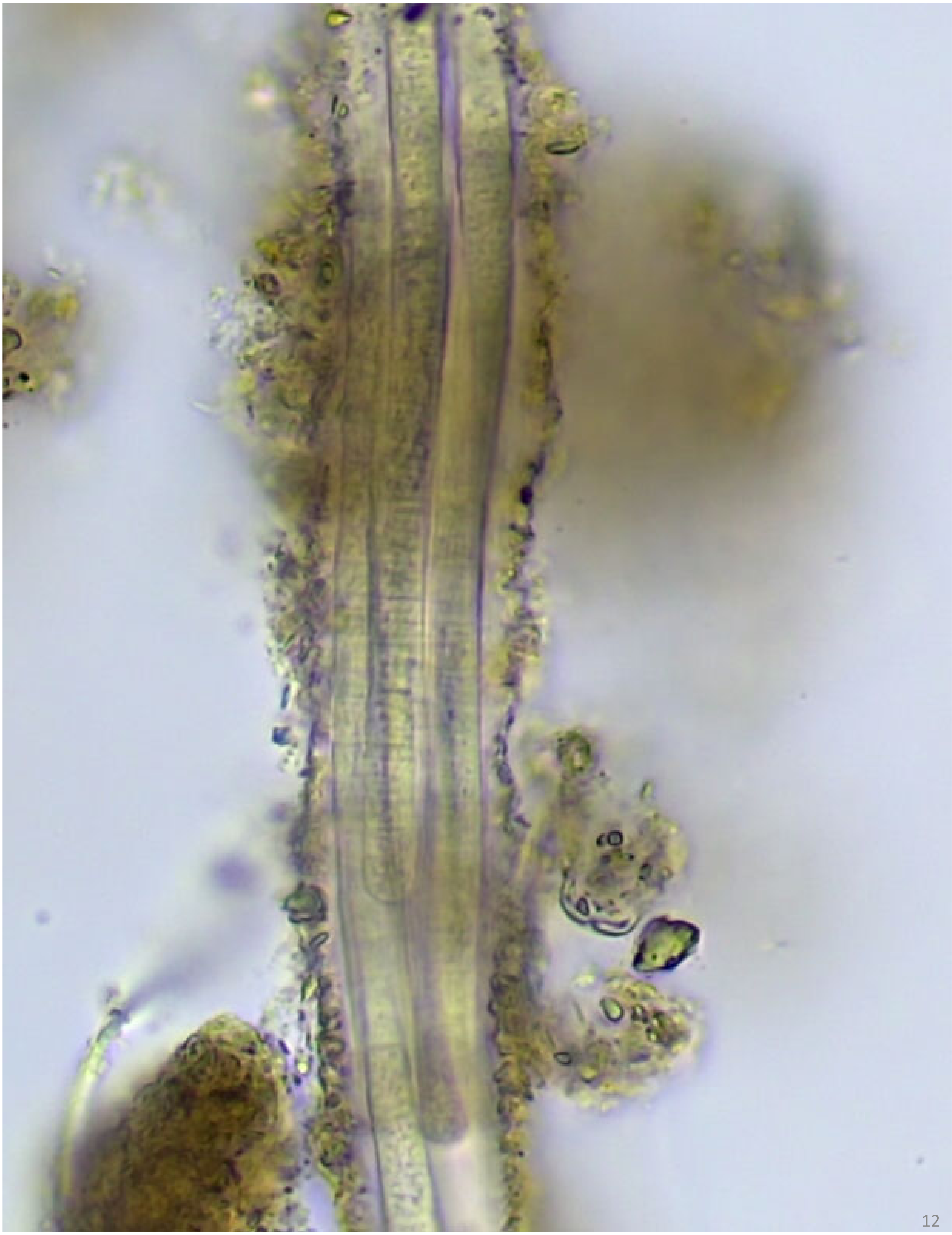

Microseira (Lyngbya) 400x. From the September 2018 NDS. Note the thick sheath and cells much wider than long. Epiphytic *Cocconeis* (diatom) colonized this filament

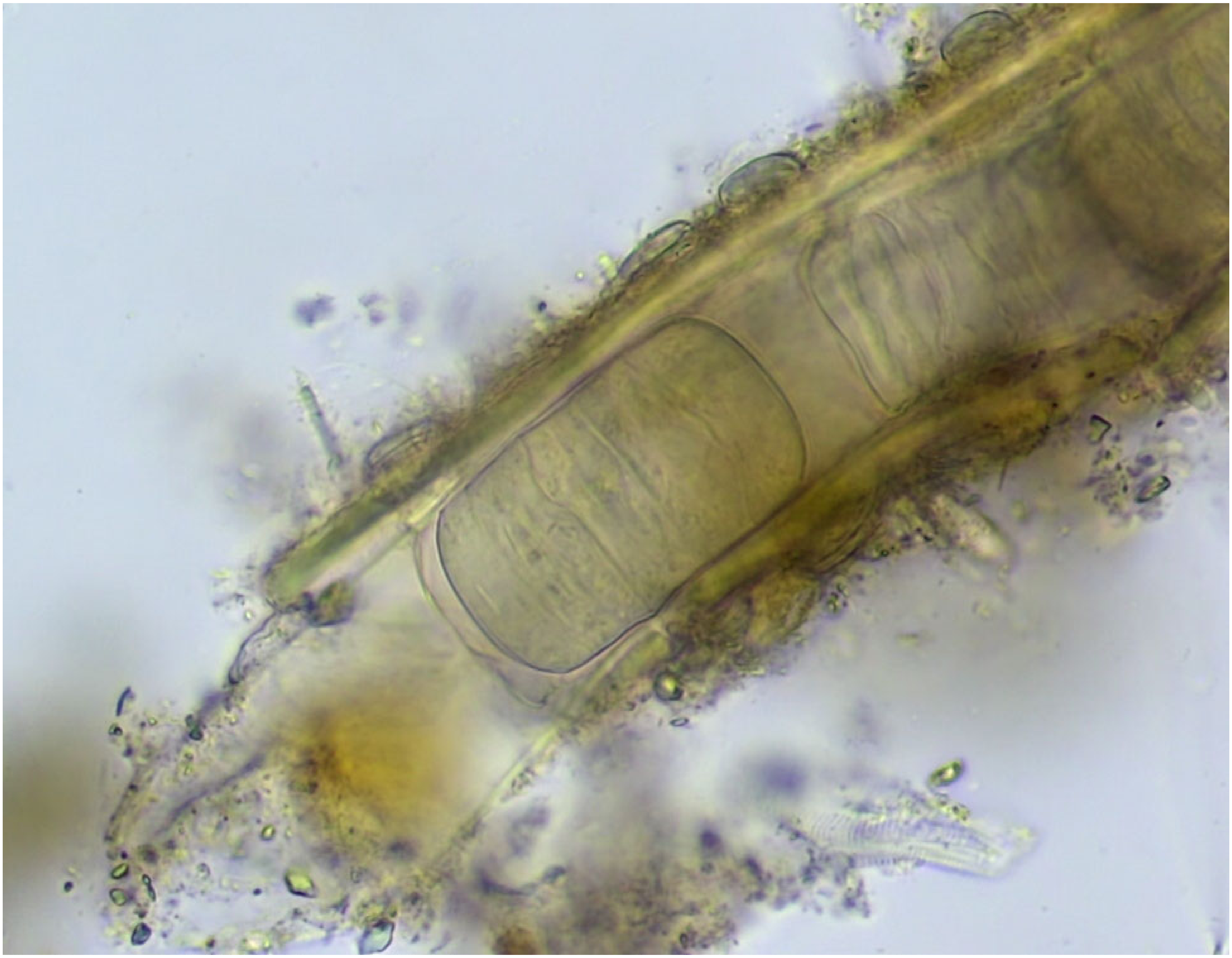

Microseira (Lyngbya) 400x. This image is of a different focal point from the previous image to show the epiphytic diatoms (*Cocconeis* sp.) and an unidentifiable thin cyanobacterium filament. From the September 2018 NDS

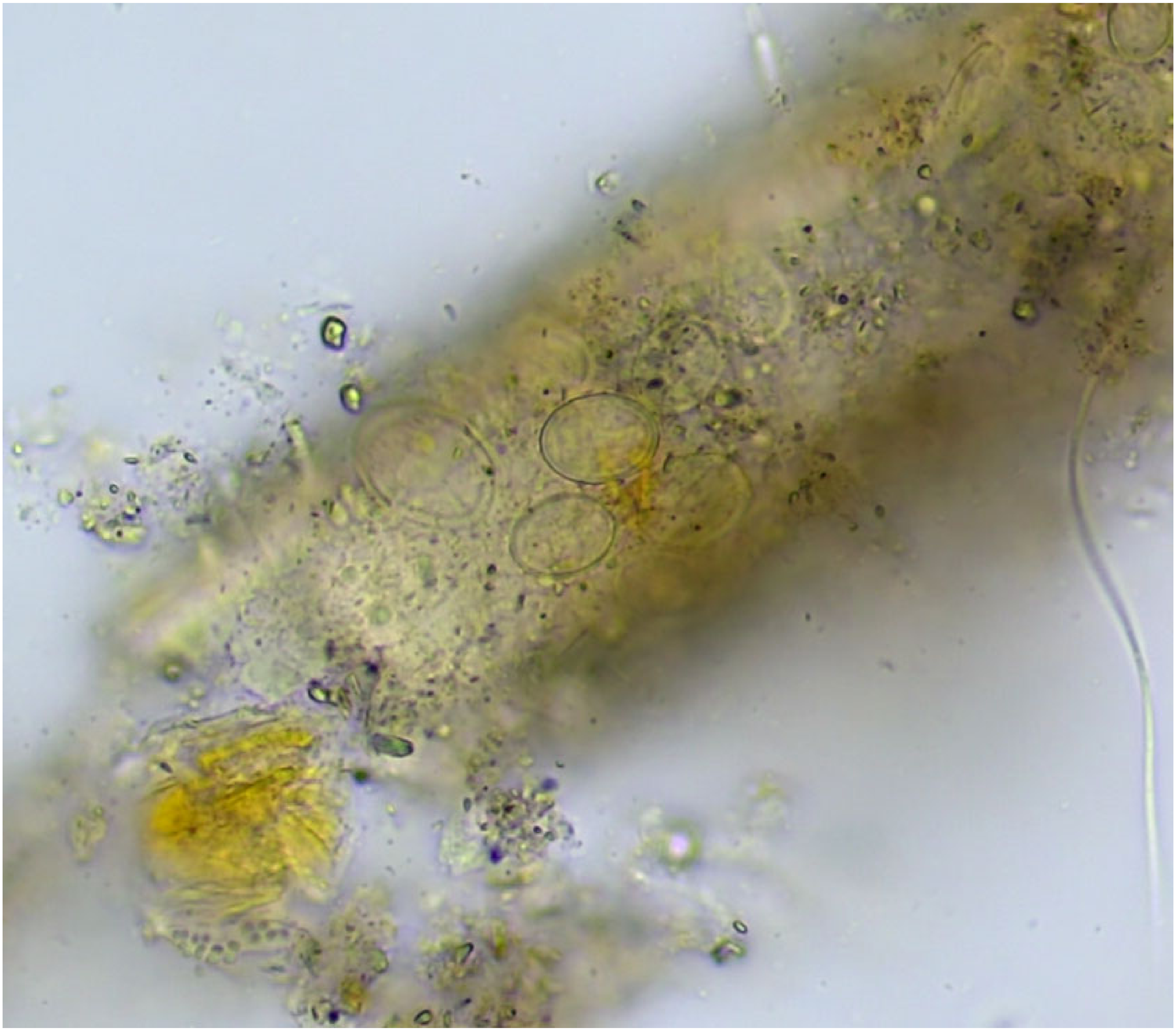

Supplement: appendix S1 [file NIHMS2040665-supplement-appendix_S1.pdf]
